# *Terminalia catappa* leaf extracts inhibited metastasis of A2058 and A375 melanoma cells *via* downregulating p-Src and β-catenin pathway *in vitro*

**DOI:** 10.3389/fphar.2022.963589

**Published:** 2022-09-27

**Authors:** Chin-Kuo Chang, Shu-Chen Chu, Jing-Yang Huang, Pei-Ni Chen, Yih-Shou Hsieh

**Affiliations:** ^1^ Institute of Medicine, Chung Shan Medical University, Taichung, Taiwan; ^2^ Institute and Department of Food Science, Central Taiwan University of Science and Technology, Taichung, Taiwan; ^3^ Department of Medical Research, Chung Shan Medical University Hospital, Taichung, Taiwan; ^4^ Department of Biochemistry, School of Medicine, Chung Shan Medical University, Taichung, Taiwan; ^5^ Clinical Laboratory, Chung Shan Medical University Hospital, Taichung, Taiwan

**Keywords:** *Terminalia catappa*, melanoma, antimetastasis, matrix metalloproteinases-2, Wnt/βcatenin pathways

## Abstract

**Background:** Melanoma is a highly aggressive, lethal, and malignant cancer. Once diagnosed early, it can be easily removed and cured with satisfaction. Although many methods such as surgery, chemotherapy, radiotherapy, and immunotherapy have been used to treat this disease at an advanced stage, the outcomes are poor. *Terminalia catappa* leaves have been shown to have various biological benefits, including antitumor activity. The specific effects and molecular mechanisms of *Terminalia catappa* leaf in treating A2058 and A375 melanoma cells *in vitro* need to be clarified.

**Methods:** The A2058 and A375 melanoma cancer cells were treated with *Terminalia catappa* leaf extracts, and then the effect of *Terminalia catappa* leaf extracts on migration and invasion was examined. The cell migration/invasion capacities of A2058 and A375 cells were investigated by a modified Boyden chamber assay. Zymography was used to clarify the activities of matrix metalloproteinases-2 and urinary type plasminogen activator. We performed a Western blot to verify the related expression of phospho-Src (Tyr416), phospho-Focal adhesion kinase (Tyr397), Vimentin, and β-catenin.

**Results:** Modified Boyden chamber assays demonstrated that treatment of *Terminalia catappa* leaf extracts significantly inhibited A2058 and A375 cell migration/invasion capacities. In the zymography results, we showed that *Terminalia catappa* leaf extracts negatively modulated the activities of matrix metalloproteinases-2 and urinary type plasminogen activator. Western blot indicated that *Terminalia catappa* leaf extracts reduced the expression of phospho-Src (Tyr416), phospho-Focal adhesion kinase (Tyr397), Vimentin, and β-catenin.

**Conclusion:** *Terminalia catappa* leaf extracts affected the antimetastasis of the A2058 and A375 melanoma cell lines by inhibiting the Focal adhesion kinase/Src interaction and Wingless-int1/β-catenin pathways *in vitro*. *Terminalia catappa* leaf extracts may serve as an effective chemopreventive agent against metastasis of melanoma cancer.

## 1 Introduction

Melanoma is a highly aggressive and deadly form of skin cancer caused by various risk factors, including environmental or genetic causes such as UV exposure and sunlight (Lo and Fisher, 2014). Melanoma with increasing incidence and mortality rate has been found in recent decades (Volkovova et al., 2012). Resistance to chemotherapy, radiation therapy and immunotherapy often results in ineffective treatment of the metastatic melanoma. Metastatic melanoma patients with a 5-year survival rate only occupied less than 20% (Sandru et al., 2014). Surgery can cure localized melanoma early, but treatment is ineffective for metastatic melanoma (Yilmaz and Christofori, 2009; Simoes et al., 2015).

During epithelial-mesenchymal transition (EMT), cancer cells aggressively infiltrate the blood vessels and lymphatic system and transfer to other organs or tissues, forming a metastatic tumor. In the process of EMT, inhibition of E-cadherin and promotion of Vimentin, N-cadherin, Focal adhesion kinase (FAK), Src, Snail, and Twist in cell junctions can be initiated at the molecular level (Lamouille et al., 2014). Changes in surface adhesion properties, cell migration and invasion, penetration into vascular or lymphoid tissues, degradation of the extracellular matrix (ECM), and infiltration into nearby tissues are among the various events triggered during cancer metastasis (Westermarck and Kahari, 1999; Gupta and Massague, 2006).

Degradation of components in the basement membrane and extracellular matrix occurs during metastasis, resulting from the combined action of multiple proteases, such as plasminogen activator (PA), matrix (MMPs), cathepsins, in tumor metastasis and plays a crucial role in the invasion transfer process (Westermarck and Kahari, 1999). The expression of matrix metalloproteinases-2 (MMP-2) has been reported to correlate with various tumors (Stetler-Stevenson, 1999). In addition to MMP, the serine protease urinary type plasminogen activator (u-PA) converts inactive plasminogen to active plasmin and elicits a cascade of the proteolytic process to degrade the extracellular matrix (Baricos et al., 1995). Enhanced activity and expression of MMP-2 have a significant impact on survival and outcomes in human malignancies (Stearns and Stearns, 1996; Talvensaari-Mattila et al., 1998; Kallakury et al., 2001; Yoshizaki et al., 2001). u-PA is present in cellular structures on the leading edge of migrating cells and regulates cell adhesion, migration, invasion, and metastasis (Duffy and Duggan, 2004). Therefore, MMP or u-PA has a controlling effect on cancer metastasis, which will be considered as a feasible method. (Bjorklund and Koivunen, 2005). FAK is overexpressed and hyperphosphorylated in multiple cancer cells, and recent reports have elucidated that this kinase is responsible for cell migration (Fan et al., 2016), survival (Skinner et al., 2016), proliferation (Balsas et al., 2017), and adhesion (Nader et al., 2016). Focal contacts are built at the ECM integration junctions that bring together the cytoskeleton and signaling proteins during cell adhesion, spread, and migration (Maziveyi and Alahari, 2017).

FAK activity can be initiated by ECM or growth factors. Previous studies have found that tyrosine phosphorylation regulates focal contact assembly and can occur rapidly (Parsons, 2003). Using the FAK mutation at Tyr397, a binding site for Src, the study verified that while downregulating cell migration, this mutation releases a pool of active Src that modulates enhanced invadopodia activity (Mousson et al., 2021). Inhibition of the FAK/Src interaction enhances the proteolytic activity of invasive melanoma cells (Mousson et al., 2021). Importantly, Src plays a control role in the expression of E-cadherin (Serrels et al., 2011). In breast cancer cells, inhibition of Src induces a reverted epithelial phenotype associated with an increase in E-cadherin and a decrease of Vimentin, and it blocks cancer cell migration (Liu and Feng, 2010). The oncogenic expression of Src in pancreatic tumor cells was also associated with a reduced expression of E-cadherin in favor of Vimentin (Nagathihalli and Merchant, 2012).

The importance of canonical Wingless-int1 (Wnt) signaling in melanoma initiation is associated chiefly with β-catenin ability to regulate the expression of a wide range of genes of the melanocyte lineage, and its involvement in the regulation of proliferation is probably related to the activation of microphthalmia-associated transcription factor (MITF) expression (Widlund et al., 2002; Larue and Delmas, 2006; Levy et al., 2006; Hoek et al., 2008; Arozarena et al., 2011; Kawakami and Fisher, 2017). β-catenin mediates the transcriptional expression of Wnt target genes (Tarapore et al., 2012; Uzdensky et al., 2013; Kahn, 2014). While the Wnt pathway activation is “turn on”, β-catenin enters the nucleus and engages DNA-bound TCF transcription factors (Behrens et al., 1996) (Molenaar et al., 1996). Numerous studies demonstrated that aberrant activation of Wnt-signaling contributed to malignant cell transformation and neoplastic proliferation with further metastatic dissemination and resistance to treatment (Ring et al., 2014; Galluzzi et al., 2019). In several studies of hepatocellular carcinoma, melanoma, and acute myeloid leukemia, FAK operated upstream of Wnt, while in prostate cancer, Wnt appeared to act upstream of FAK. Furthermore, a study of renal carcinoma revealed that Wnt and FAK might work simultaneously in promoting cancer progression (Worthmuller and Ruegg, 2020). The depletion of FAK in hepatocytes blocked tumor proliferation and prolonged survival in a c-Met/β-catenin-driven hepatocellular carcinoma (HCC) mouse model (Shang et al., 2015), suggesting that FAK is required for c-Met/β-catenin-driven hepatocarcinogenesis. Another study showed that FAK and β-catenin synergistically induced HCC in mice (Shang et al., 2019) and that FAK elicited Wnt/β-catenin signaling, activated cancer stem cell (CSC) traits, and initiated tumorigenicity in HCC cells (Fan et al., 2019).

The *Terminalia catappa* leaf is biologically active, including antioxidants (punicalagin, punicalin, chebulic acidcoumaric acid, benzoic acid, terfluvina A and B, and their derivatives) (Chen and Li, 2006; Kinoshita et al., 2007), antidiabetic (beta - carotene) (Anand et al., 2015), anticancer (punicalagin) (Naitik et al., 2012), antiviral (ellagic acid) (Tan et al., 1991), anti-inflammatory (triterpene acids, especially ursolic acid and its derivatives) (Fan et al., 2004), antibacterial (flavonoids and flavanols) (Kloucek et al., 2005; Shinde et al., 2009), and hepatoprotective activity (punicalagin, punicalin) (Kinoshita et al., 2007). *Terminalia catappa* leaf extracts (TCE) effectively inhibits phosphorylation of the ERK1/2 signaling pathway by negatively regulating the activity of the DNA binding factor SP-1 and NF-κB DNA-binding activity, thereby inhibiting u-PA and inhibiting metastasis (Yeh et al., 2014). TCE had an antitumor influence in Ehrlich ascites carcinoma (EAC)-bearing mice with EAC (Pandya et al., 2013) by modulating Lipid Hydroperoxide (LPO) and reinforcing the antioxidant defense system. In this report, our goal was to study the efficacy of TCE on antimetastasis of the A2058 and A375 melanoma cell line and related mechanisms that will contribute to humans.

## 2 Materials and methods

### 2.1 Preparation of TCE

*Terminalia catappa* leaves were obtained from a traditional herbal medicine store in Taichung. *Terminalia catappa* ethanol extracts were prepared by initial condensation and lyophilization as previously described (Chu et al., 2007). Briefly, 100 g of air-dried leaves were boiled at 70°C with 500 ml of 50% ethanol for 24 h. We removed the solvent by using a vacuum rotary evaporator. These fractions were then lyophilized and stored at −20°C. Next, we dissolved the TCE powder in 50% dimethyl sulfoxide (DMSO) to achieve an indicated concentration with the highest DMSO concentration of less than 0.1%.

### 2.2 Cell and cell culture

We obtained A2058 and A375 human melanoma cell lines from the Bioresource Collection and Research Center (BCRC, Hsinchu, Taiwan) and cultured them in Dulbecco’s Modified Eagle Medium (DMEM). All cell cultures were kept in a humidified atmosphere at 37°C containing 5% CO_2_.

### 2.3 Determination of cell viability (MTT assay)

TCE cytotoxicity was evaluated using an MTT colorimetric assay (Sigma) (Mosmann, 1983; Yang et al., 2008) to analyze cell viability. After seeding in 24-well plates at a density of 5 × 10^4^ cells/well, A2058 cells, A375 and Hs68 cells were treated with TCE, respectively, at a concentration between 0 and 100 μg/ml at 37°C for 24 h. The medium was removed after 24 h of incubation, and the two cells were washed with phosphate-buffered saline (PBS) 3 times and then incubated with 20 μL of MTT (5 mg/ml) (Sigma Chemical Co., St. Louis, MO, United States) for 4 h. The number of viable cells per plate was measured spectrophotometrically at 563 nm after solubilization with isopropanol.

### 2.4 Determination of urinary type plasminogen activator and matrix metalloproteinase 2 by zymography

MMP-2 in a conditioned medium were examined using gelatin zymography protease assays, as previously described (Chu et al., 2007). To prepare samples, we used a standard sodium dodecyl sulfate gel loading buffer containing 0.01% SDS without β-mercaptoethanol without boiling or reduction. The collected samples were then subjected to 8% sodium dodecyl sulfate polyacrylamide gel electrophoresis (SDS-PAGE) (0.75-mm; acrylamide/bis-acrylamide 30/1.2; containing 0.1% gelatin; Sigma). Electrophoresis was performed for 3 h at 140 V in an OWL P-1 apparatus (Alpha Multiservices, Inc., Contoe, TX, United States). After electrophoresis, we used 100 ml of distilled water containing 2% Triton X100 (Sigma) to wash the gels twice. The gels were then incubated in reaction buffer (40 mM Tris-HCl, pH 8.0, 10 mM CaCl2, 0.02% NaN3; Sigma) at 37°C for 12 h, stained with Coomassie brilliant blue R250 and destained with methanol/acetic acid/water (50/75/875, v/v/v; Sigma). The activity of u-PA was verified as previously described (Chu et al., 2007). 2% w/v casein and 20 μg/ml plasminogen (Sigma) were added to 8% SDS-PAGE gels. Electrophoresis and zymography were performed for gelatin zymography.

### 2.5 Wound healing migration assay

We used silicon culture inserts (Ibidi, Munchen, Germany) with two individual wells. For cell seeding, a wound closure seeding model was managed to determine whether TCE could suppress A2058 cell migration and A375 cell migration, respectively. Each insert was placed in a culture plate, and A2058 or A375 were seeded with 1.5 × 10^4^ cells/well and grown to form a confluent and homogeneous layer. After incubation for 24 h, the culture insert was removed, and the wound zone could be found. The medium was removed and replaced with DMEM containing 1% FBS, and then TCE was added. We incubated the two cells at 37°C and photographed the movement of the cells in the wound zone at 0 h, 24 h, and 48 h using a phase-contrast microscope (×40).

### 2.6 Cell invasion and migration assays

To validate the motility and invasion, a Boyden chamber assay (Neuro Probe, Cabin John, MD, United States) with a pore size of 8 μm for the migration assay and a matrix barrier of 10 μg/ml Matrigel (BD Biosciences, MA, United States) for the invasion assay, respectively. First, we treated A2058 and A375 cells with TCE at different concentrations (0, 25, 50, and 100 μg/ml) for 24 h. The surviving cells and seeded in a Boyden chamber with 1.5 × 10^4^ cells/well in a serum-free medium and incubated at 37°C for 12 h. For the invasion assay, 10 μg/ml Matrigel^®^ (25 mg/50 ml; BD Biosciences, Bedford, MA, United States) was applied to 8-μm-pore-size polycarbonate membrane filters (Neuro Probe, Cabin John, MD, United States), and the bottom chamber was contained with a traditional medium. Following incubation, the filters were air-dried for 5 h in a laminar flow hood. We fixed invading cells with methanol and stained them with Giemsa (Sigma). We counted cell numbers with a light microscope (CKX41; Olympus), and the migration assay was practiced as described for the Matrigel invasion assay, without the coating (Chen et al., 2006).

### 2.7 Preparation of total cell lysates

For the preparation of total cell lysates, cells were rinsed twice with PBS and scraped with 0.2 ml of cold RIPA buffer containing protease inhibitor cocktail, then vortexed at 4°C for 10 min. Cell lysates were subjected to 13,000 rpm centrifugation for 10 min at 4°C, and the insoluble pellet was discarded. Briefly, harvested cells were scraped and lysed with buffer A (10 mM HEPES, 10 mM KC1, 0.1 mM EDTA, 1.5 mM MgCl_2_, 0.2% NP40, 1 mM DTT, and 0.5 mM phenylmethylsulfonylfluoride), followed by vortexing to shear the cytoplasmic membranes were collected by centrifugation at 13,000 rpm for 30 s at 4°C. Finally, we performed a Bradford assay (Bradford, 1976) to verify the concentration of total cell lysates.

### 2.8 Western blot analysis

A 6 cm dish with seeded cells was incubated for 24 h. TCE was added with an indicated concentration (0, 25, 50, and 100 μg/ml) for 24 h. We used the PRO-PREP protein extraction solution (iNtRON), containing the protease inhibitor, to collect total cell lysates. Subsequently, the cell extracts were microcentrifuged at 13,000 rpm for 30 min at 4°C, and the supernatants were obtained. Cell lysates were separated on a 10% polyacrylamide gel and transferred to a nitrocellulose membrane. And the blot was incubated with 5% non-fat milk in PBS for 1 h to block non-specific binding and then the corresponding antibodies overnight against GAPDH (Santa Cruz Biotechnology Inc., Santa Cruz, CA), Vimentin (Santa Cruz Biotechnology Inc., Santa Cruz, CA), p-Src (Tyr416) (Cell Singling Technology, Inc., Danvers, MA, United States), t-Src (Cell Singling Technology, Inc., Danvers, MA, United States), p-FAK (Tyr397) (Cell Singling Technology, Inc., Danvers, MA, United States), t-FAK (Cell Singling Technology, Inc., Danvers, MA, United States) and β-catenin (Gene Tex. Inc. Irvine, CA, United States). The cells were then incubated at 37°C with an appropriate peroxidase-conjugated secondary antibody for 1 h. Then we used enhanced chemiluminescence (ECL) commercial kit (Amersham Biosciences) to obtain signals and quantitated the relative photographic density by scanning the photographic negatives on a gel documentation and analysis system (AlphaImager 2000; Alpha Innotech Corporation, San Leandro, CA, United States) (Yang et al., 2008).

### 2.9 Statistical analysis

Data are indicated as mean ± SD, and each experiment was performed in triplicate. Statistical significances were analyzed by two-way analysis of variance (ANOVA) with post hoc Tukey’s test.

The *p*-value <0.05 was indicated as statistically significant.

## 3 Results

### 3.1 The influence of TCE on the viability of A2058, A375 and Hs60 cells

**FIGURE 1:**
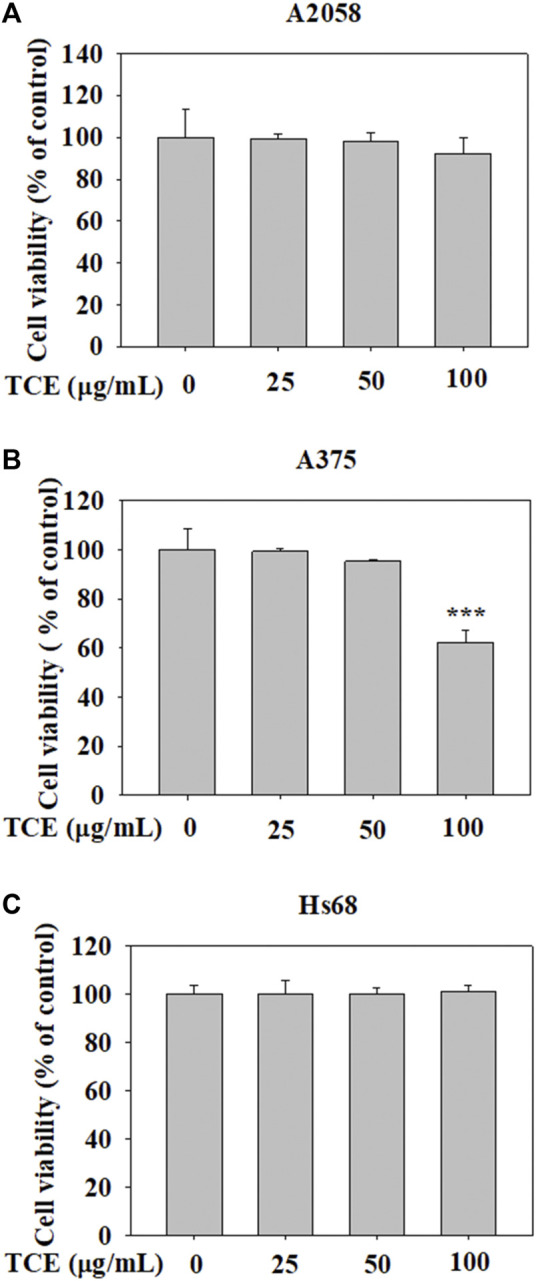
The effects of TCE cell viability. **(A)** A2058, **(B)** A375 cells and **(C)** normal dermal fibroblastHs68 were treatedwith TCE (0, 25, 50, 100 μg/ml), respectively, for 24 h by MTT assay. The values represent the means ± SD of at least three independent experiments. Comparisons were performed using two-way ANOVA with post hoc Turkey’s test (***p < 0.05).

The cytotoxic effects of TCE at various concentrations (0–100 μg/ml) on A2058 cells and A375 cells are shown in Figures 1A,B. The assay showed that even at the highest concentration of 100 μg/ml. TCE did not alter the viability of A2058 and had a little impact on A375 cells compared to controls. In addition, the effect of TCE on the cell viability of normal Hs60 fibroblasts was also investigated. As shown in Figure 1C, TCE did not alter the viability of Hs60 cells.

### 3.2 Reduced effect of TCE on the activity of MMP-2

**FIGURE 2:**
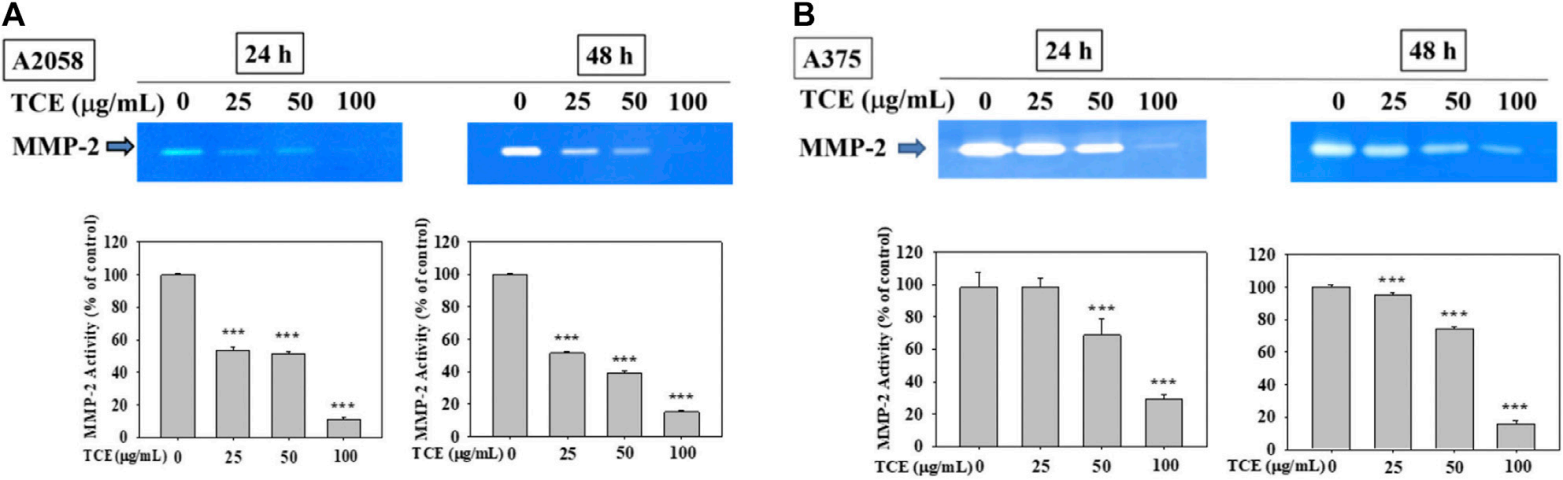
Effects of TCE on the activity of MMP-2. A2058 cells and A375 were treated with (0, 25, 50, 100 μg/ml) of TCE for 24 h and 48 h, respectively, then subjected to gelatin and casein zymography to analyze the activities of MMP-2 **(A**, **B)**. The values represented the means ± SD of at least three independent experiments. Comparisons were performed using two-way ANOVA with post hoc Turkey’s test (***p < 0.05).

As shown in Figures 2A,B, TCE treatment in A2058 and A375 cells for 24 h and 48 h can reduce the activity of the MMP-2 protein, and this reduction was dose-dependent.

### 3.3 Reduced effect of TCE on the activities of u-PA

**FIGURE 3:**
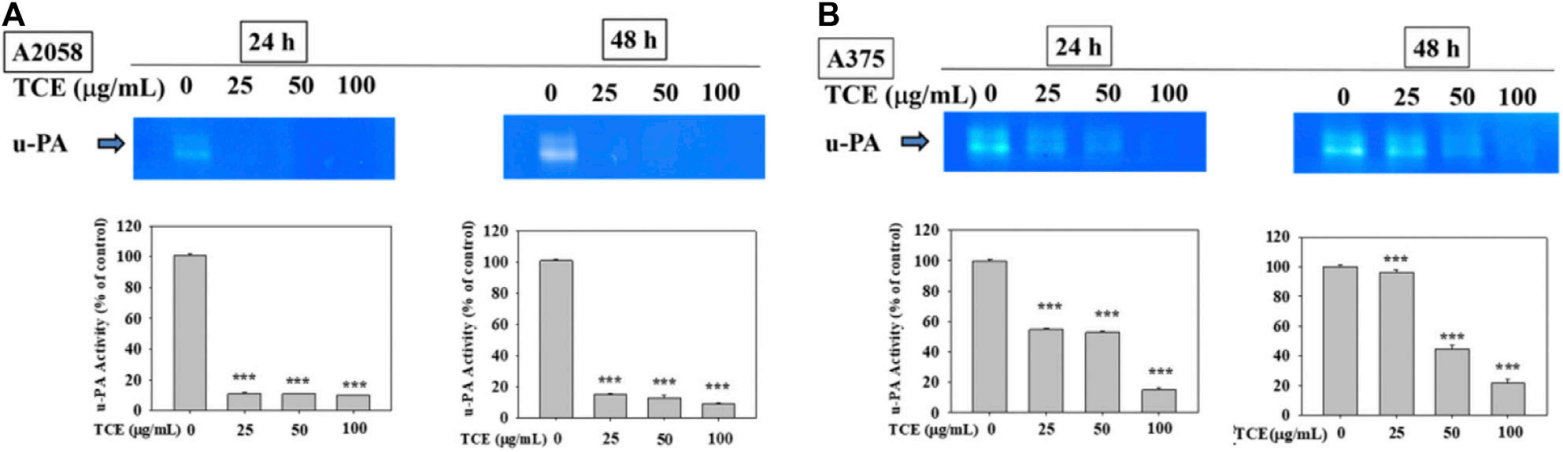
Effects of TCE on the activity of u-PA. A2058 cells and A375 were treated with (0, 25, 50, 100 μg/ml) of TCE for 24 h and 48 h, respectively, then subjected to gelatin and casein zymography to analyze the activities of u-PA **(A**, **B)**. The values represented the means ± SD of at least three independent experiments. Comparisons were performed using two-way ANOVA with post hoc Turkey’s test (***p < 0.05).

Figures 3A,B showed that TCE treatment in A2058 and A375 cells for 24 h and 48 h can suppress the activity of the u-PA protein, and this suppression was dose-dependent.

### 3.4 Inhibitory effect on the motility of A2058 and A375 cells by TCE

**FIGURE 4:**
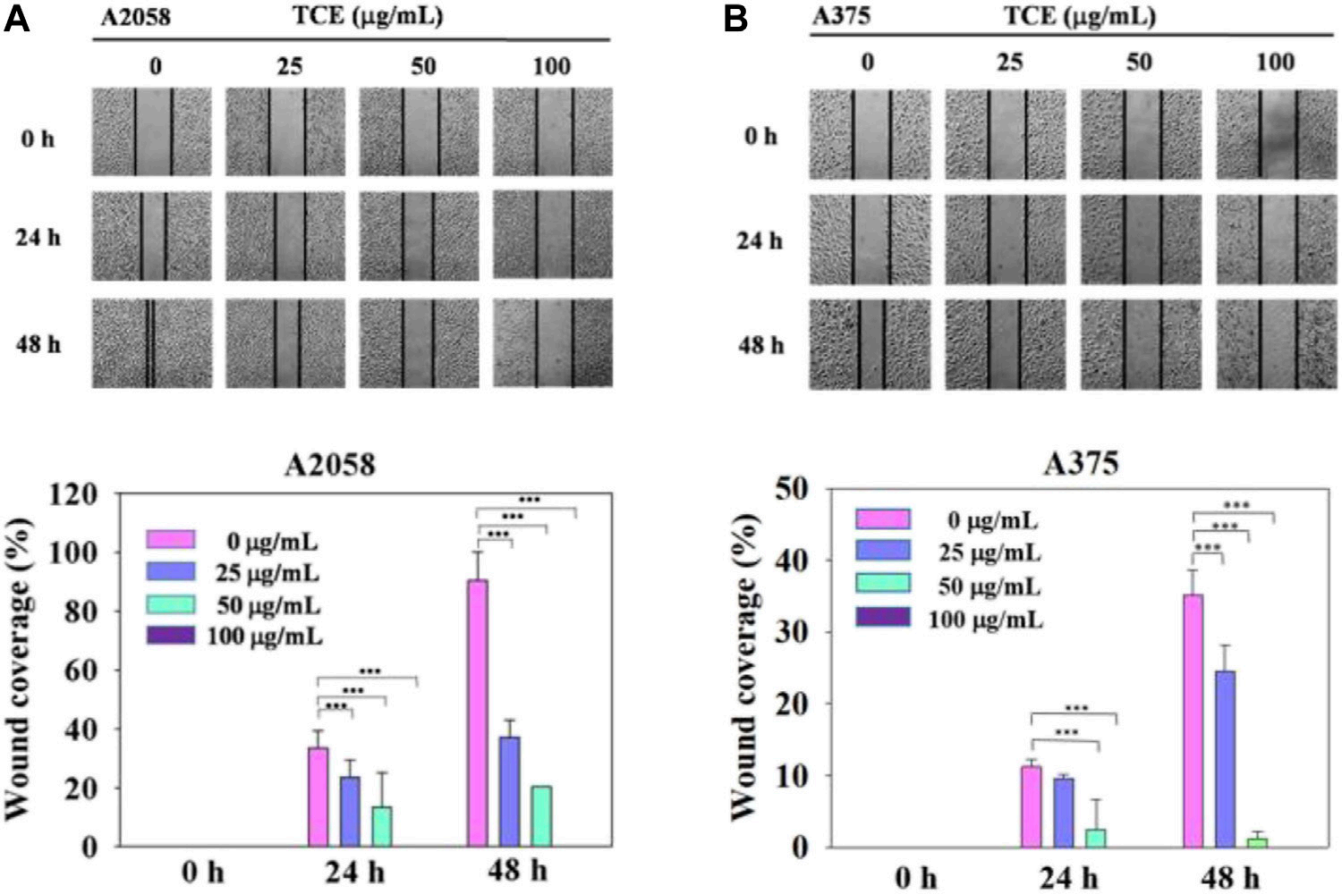
The effects of TCE on the cell migration of A2058 and A375 cells. **(A,B)** Photographs showed wound closure of A2058 and A375 cells treated with TCE (0, 25, 50, 100 μg/mL) and 0, 24, 48 h. Cell wound healing assay was performed as described in the Materials and Methods. % Wound coverage was determined by the rate of cells moving forward the scratched area upon time. The values of percentagewound closure ± SD of at least three independent experiments. Comparisons were performed using two-way ANOVA with post hoc Turkey's test (***p < 0.05).

Regarding the cell migration, we observed that after 24 h at concentration 0 μg/ml, 25 μg/ml, 50 μg/ml with TCE, the A2058 cells migrated and covered approximately 15%–35% of the wound area quantified in time zero for all abutment discs, the A375 cells 2%–13%. And after 48 h at concentration 0 μg/ml, 25 μg/ml, 50 μg/ml with TCE, the A2058 cells migrated and covered approximately 21%–97% of the wound area quantified in time zero for all abutment discs, the A375 cells 1%–32%. Lower migration was observed on A375 when compared to A2058. Initial wound edges marked initial cell migration and were used to identify the decrease in wound width throughout the experiment. Migration distances were showed separately during periods 0–24 h (migration during first 24 h period) and 0–48 h (during second 24 h period). On the two images recording period after wounding (0–24 h) (0–48 h) at 0 μg/ml, 25 μg/ml, 50 μg/ml 100 μg/ml with TCE, significant differences migration distance were found for A2058 discs when compared to the control (Figure 4A, ***p < 0.05), whilst no difference in motility was found on A375 discs after wounding (0–24 h) at 25 μg/ml concentration with TCE (Figure 4B, ***p < 0.05). Experiments are showed in triplicates (mean ± SD, ***p < 0.05).

### 3.5 Inhibitory effect on migration and invasion of A2058 and A375 cells by TCE

**FIGURE 5:**
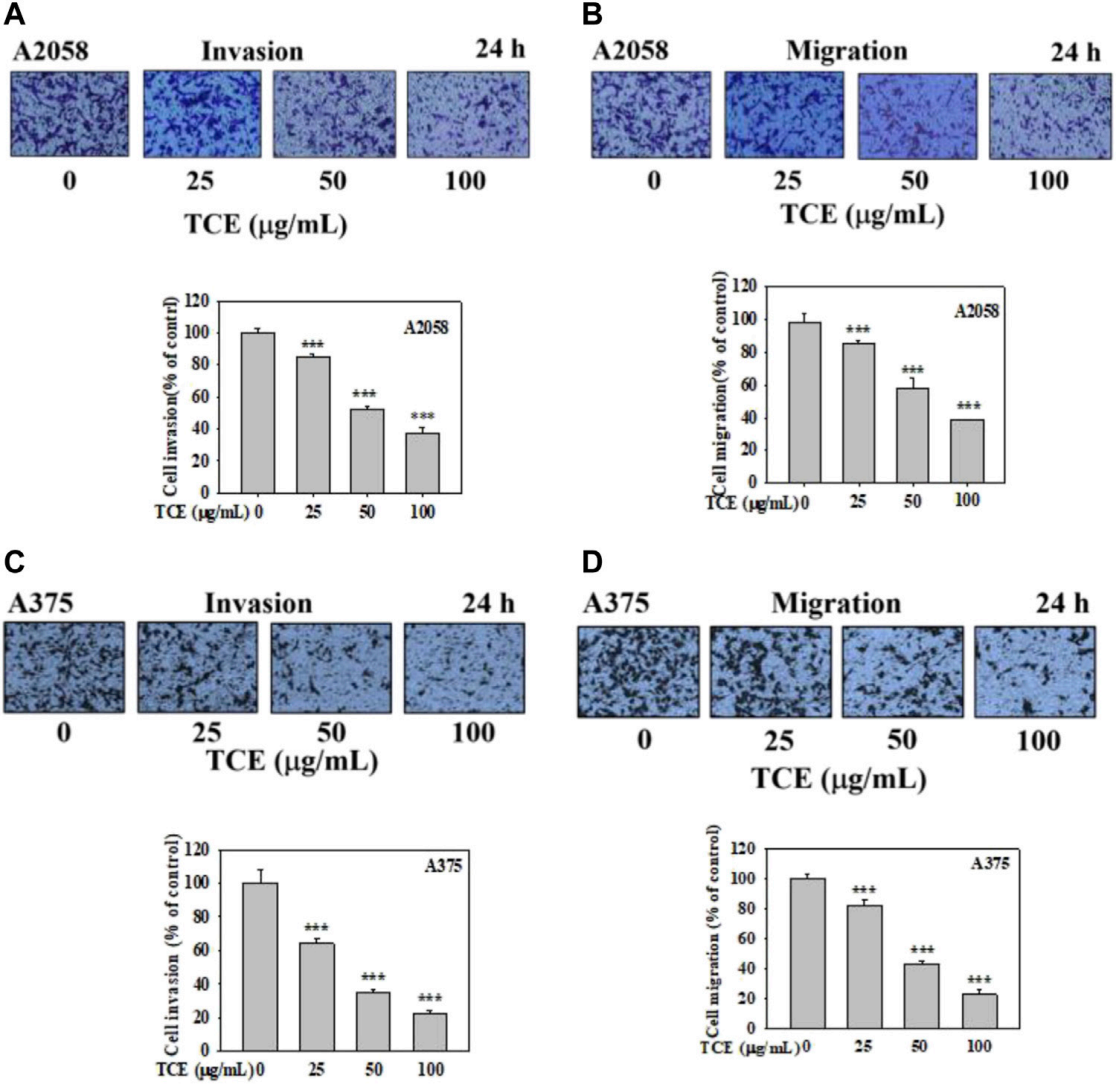
Effects of TCE on cell invasion and migration of A2058 and A375 cells. After being treated with TCE at a concentration of (0, 25, 50, 100 μg/mL) for 24 h, cell invasion and migration assay were then performed as described in the Materials and Methods. Representative images of A2058 and A375 cells on the lower side of membrane at different time points in the **(A,C)** invasion and **(B,D)** migration assay. A representative number of invading cells through the Matrigel and membrane were counted under the microscope for 10 random fields at a 3,200 magnification. The results were statistically evaluated using two-way ANOVA with post hoc Turkey's test (***p < 0.05). The results from 3 repeated and separate experiments were similar.

It demonstrated that TCE significantly suppressed the invasion of A2058 and A375 cells in a concentration-dependent manner, only 38% and 20% remained after treatment of 100 μg/ml with TCE, respectively (Figures 5A,C), 38% and 22% left after cell migration (Figures 5B,D).

### 3.6 Down-regulated effect of the vimentin, p-FAK (Tyr397), p-Src (Tyr416), and β-catenin pathways by TCE

**FIGURE 6:**
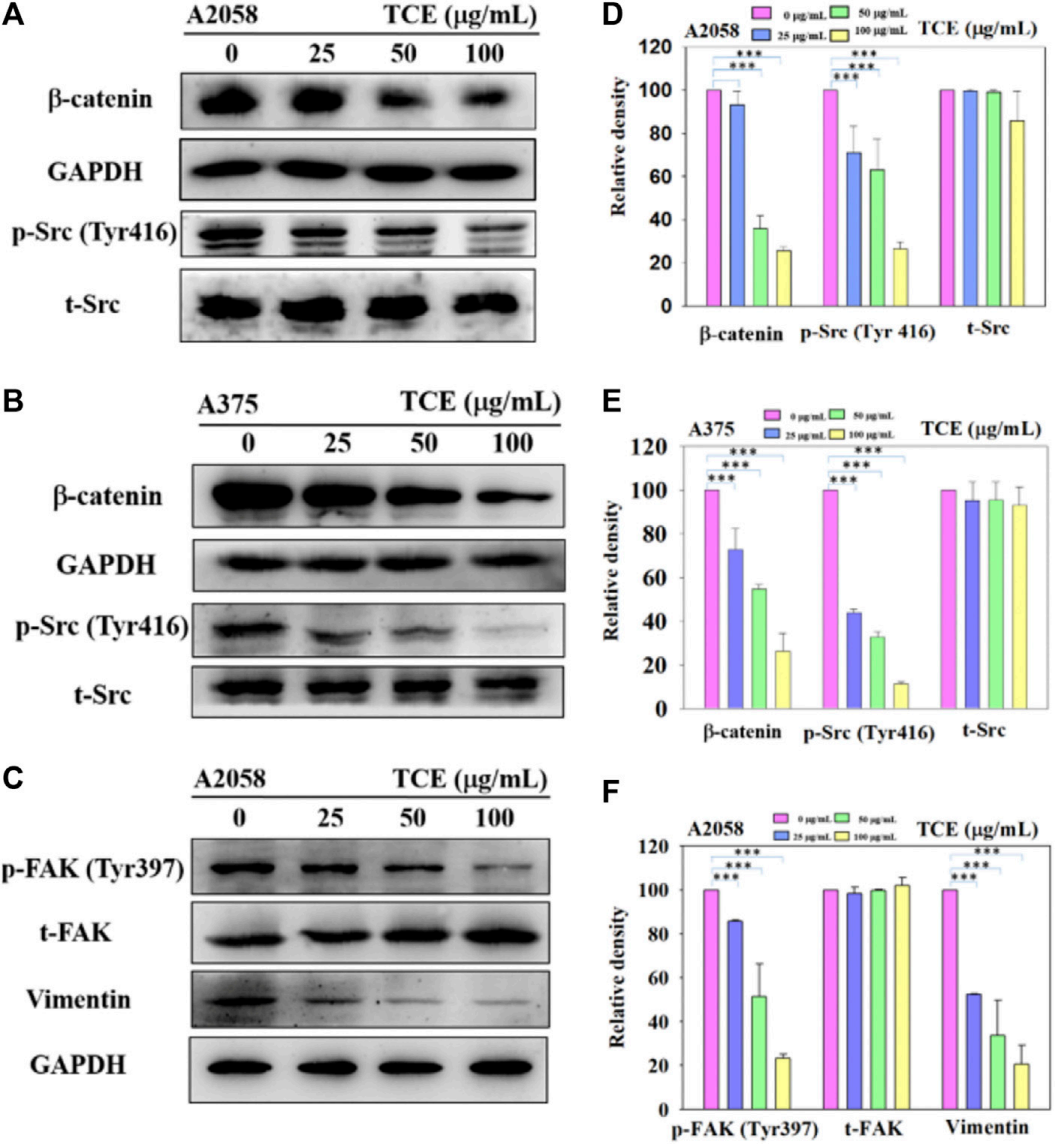
A2058 and A375 cell lines were pretreated with TCE and then incubated for 24 h. The cells lysates were subjected to SDS-PAGE followed by western blotswith anti-β-catenin, anti-p-Src (Tyr416), anti-t-Src, anti-GAPDH **(A**, **B)** and anti-Vimentin, anti-p-FAK (Tyr397), anti-t-FAK, anti-GAPDH for A2058 **(C)**, as described in Methods Section 2.8. The bar graph shows the statistical results of protein expression **(D**–**F)**. The values represented the means ± SD of at least three independent experiments. Comparisons were performed using two-way ANOVA with post hoc Turkey’s test (***p < 0.05).

Since the inhibitory effect of TCE on cell migration/invasion and proteinases has been revealed, the effects of TCE on the β-catenin pathways were investigated by Western blot to clarify the underlying mechanisms. The Western blot results indicated that TCE could reduce the phosphorylation of β-catenin and p-Src (Tyr416) (Figures 6A,B) in A2058 and A375 cells. Additionally, TCE could also inhibit the expressions of Vimentin and p-FAK (Tyr397) in A2058 cells (Figure 6C).

## 4 Discussions

In the past, our laboratory has also studied TCE in other cancer metastases (Chu et al., 2007; Yang et al., 2010; Yeh et al., 2012; Chung et al., 2021). We demonstrated that TCE negatively affected metastasis in various cancers, including hepatocellular carcinoma, oral cancer, human glioblastoma, and lung cancer. To explore in-depth the effect and mechanism of TCE in anticancer metastasis, we used various verification methods to analyze the actual biological results of TCE on the antimetastasis of melanoma *in vitro*. First, we found that TCE could reduce the expression of MMP-2 and u-PA, respectively, for 24 and 48 h in the zymography assay (Figure 2 and Figure 3). As we know, MMP-2, and u-PA pose an essential role during metastasis. Effective down-regulation of the function of MMP-2 and u-PA by TCE led to the depletion of ECM (Baricos et al., 1995). It showed that the down-regulated expression of MMP-2 lost its fundamental ability to facilitate metastasis activity under the effect of TCE. In the movement wound healing assay, TCE significantly hindered the moving ability of the A2058 and A375 cell lines at a concentration of 50 μg/ml and 100 μg/ml with TCE (Figure 4). Whether TCE could suppress the invasion and migration of A2058 and A375 melanoma cell lines, which we expected to elucidate, should be further addressed. In the invasion and migration assay, we verified that the A2058 and A375 cell lines were suppressed and unable to invade and migrate successfully (Figure 5).

In metastasis, u-PA could convert plasminogen to plasmin, eliciting MMP-2 to degrade ECM (Chen et al., 2011). u-PA interacted with integrin-β1 and then mediated FAK to increase the downstream cascade of the metastatic mechanism. Inhibited expression of u-PA decreased the degradation activity of ECM and consequently obstructed the signaling pathway of metastasis. FAK in cells plays a pivotal role in assembling various proteins to respond further once the metastatic mechanism is initiated (Sulzmaier et al., 2014). The FAK-Src complexes phosphates Paxillin Tyr118, facilitating the combination of ERK2 and paxillin. Excellular regulated protein kinases 2 (ERK2) phosphating paxillin triggers FAK combining with paxillin and activates FAK. FAK Tyr925 was phosphated by Src and influenced the generation of paxillin and vinculin complexes (Mitra and Schlaepfer, 2006). Furthermore, Rho/Rac/Cdc42 or MAPK, which mediates cell migration and morphology, can be enhanced by interacting upstream signaling pathways between FAK Tyr925 and Src. In Western blot, we discovered that p-Src was inhibited in our study. The β-catenin pathway was also negatively regulated. After TCE treatment, the Src and β-catenin signaling pathways were hindered in the A2058 and A375 melanoma cell lines (Figure 5). TCE had the effect of tempering the FAK/Src interaction and β-catenin signaling pathway from the investigation of Vimentin, p-FAK (Tyr397) and p-Src (Tyr416), and β-catenin which were negatively regulated in Western blot (Figure 6).

The expression of p-FAK (Tyr397) and p-Src (Tyr416) protein was suppressed in Western blot (Figure 6). Previous studies showed that melanoma cells might be hindered in accelerating invadopodia activity, and subsequently, Vimentin was suppressed (Liu and Feng, 2010; Nagathihalli and Merchant, 2012; Mousson et al., 2021). Involvement in regulating melanocyte proliferation, β-catenin is most likely associated with the activation of MITF expression (Larue and Delmas, 2006). Wnt and FAK might simultaneously encourage renal carcinoma progression in a previous study (Shang et al., 2015). Interestingly, we found that TCE inhibited the expression of FAK and β-catenin in our research. It may suggest that TCE could influence the effect of metastasis of A2058 and A375 melanoma cell lines.

In conclusion, our study raveled about the possibility of adjuvant treatment of melanoma in the future. Our study demonstrated that TCE had an effect on antimetastasis in the A2058 and A375 melanoma cell line *in vitro*.

## Data Availability

The raw data supporting the conclusions of this article will be made available by the authors, without undue reservation.

## References

[B1] Anand A. V. Divya N. Kotti P. P. (2015). An updated review of Terminalia catappa. Pharmacogn. Rev. 9, 93–98. 10.4103/0973-7847.162103 26392705 PMC4557241

[B2] Arozarena I. Bischof H. Gilby D. Belloni B. Dummer R. Wellbrock C. (2011). In melanoma, beta-catenin is a suppressor of invasion. Oncogene 30, 4531–4543. 10.1038/onc.2011.162 21577209 PMC3160497

[B3] Balsas P. Palomero J. Eguileor A. Rodriguez M. L. Vegliante M. C. Planas-Rigol E. (2017). SOX11 promotes tumor protective microenvironment interactions through CXCR4 and FAK regulation in mantle cell lymphoma. Blood 130, 501–513. 10.1182/blood-2017-04-776740 28533307

[B4] Baricos W. H. Cortez S. L. el-Dahr S. S. Schnaper H. W. (1995). ECM degradation by cultured humanmesangial cells is mediated by a PA/plasmin/MMP-2 cascade. Kidney Int. 47, 1039–1047. 10.1038/ki.1995.150 7540230

[B5] Behrens J. von Kries J. P. Kuhl M. Bruhn L. Wedlich D. Grosschedl R. (1996). Functional interaction of beta-catenin with the transcription factor LEF-1. Nature 382, 638–642. 10.1038/382638a0 8757136

[B6] Bjorklund M. Koivunen E. (2005). Gelatinase-mediated migration and invasion of cancer cells. Biochim. Biophys. Acta 1755, 37–69. 10.1016/j.bbcan.2005.03.001 15907591

[B7] Bradford M. M. (1976). A rapid and sensitive method for the quantitation of microgram quantities of protein utilizing the principle of protein-dye binding. Anal. Biochem. 72, 248–254. 10.1006/abio.1976.9999 942051

[B8] Chen P. N. Hsieh Y. S. Chiang C. L. Chiou H. L. Yang S. F. Chu S. C. (2006). Silibinin inhibits invasion of oral cancer cells by suppressing the MAPK pathway. J. Dent. Res. 85, 220–225. 10.1177/154405910608500303 16498067

[B9] Chen P. S. Li J. H. (2006). Chemopreventive effect of punicalagin, a novel tannin component isolated from Terminalia catappa, on H-ras-transformed NIH3T3 cells. Toxicol. Lett. 163, 44–53. 10.1016/j.toxlet.2005.09.026 16242868

[B10] Chen R. X. Xia Y. H. Xue T. C. Zhang H. Ye S. L. (2011). Down-regulation of osteopontin inhibits metastasis of hepatocellular carcinoma cells via a mechanism involving MMP-2 and uPA. Oncol. Rep. 25, 803–808. 10.3892/or.2010.1116 21174062

[B11] Chu S. C. Yang S. F. Liu S. J. Kuo W. H. Chang Y. Z. Hsieh Y. S. (2007). *In vitro* and *in vivo* antimetastatic effects of Terminalia catappa L. leaves on lung cancer cells. Food Chem. Toxicol. 45, 1194–1201. 10.1016/j.fct.2006.12.028 17303298

[B12] Chung H. H. Hsieh M. J. Hsieh Y. S. Chen P. N. Ko C. P. Yu N. Y. (2021). The inhibitory effects of Terminalia catappa L. Extract on themigration and invasion of human glioblastoma multiforme cells. Pharm. (Basel) 14, 1183. 10.3390/ph14111183 PMC8620508 34832965

[B13] Duffy M. J. Duggan C. (2004). The urokinase plasminogen activator system: A rich source of tumour markers for the individualised management of patients with cancer. Clin. Biochem. 37, 541–548. 10.1016/j.clinbiochem.2004.05.013 15234235

[B14] Fan T. Chen J. Zhang L. Gao P. Hui Y. Xu P. (2016). Bit1 knockdown contributes to growth suppression as well as the decreases ofmigration and invasion abilities in esophageal squamous cell carcinoma via suppressing FAK-paxillin pathway. Mol. Cancer 15, 23. 10.1186/s12943-016-0507-5 26956728 PMC4782287

[B15] Fan Y. M. Xu L. Z. Gao J. Wang Y. Tang X. H. Zhao X. N. (2004). Phytochemical and antiinflammatory studies on Terminalia catappa. Fitoterapia 75, 253–260. 10.1016/j.fitote.2003.11.007 15158981

[B16] Fan Z. Duan J. Wang L. Xiao S. Li L. Yan X. (2019). PTK2 promotes cancer stem cell traits in hepatocellular carcinoma by activating Wnt/β-catenin signaling. Cancer Lett. 450, 132–143. 10.1016/j.canlet.2019.02.040 30849480

[B17] Galluzzi L. Spranger S. Fuchs E. Lopez-Soto A. (2019). WNT signaling in cancer immunosurveillance. Trends Cell Biol. 29, 44–65. 10.1016/j.tcb.2018.08.005 30220580 PMC7001864

[B18] Gupta G. P. Massague J. (2006). Cancer metastasis: Building a framework. Cell 127, 679–695. 10.1016/j.cell.2006.11.001 17110329

[B19] Hoek K. S. Eichhoff O. M. Schlegel N. C. Dobbeling U. Kobert N. Schaerer L. (2008). *In vivo* switching of human melanoma cells between proliferative and invasive states. Cancer Res. 68, 650–656. 10.1158/0008-5472.CAN-07-2491 18245463

[B20] Kahn M. (2014). Can we safely target the WNT pathway? Nat. Rev. Drug Discov. 13, 513–532. 10.1038/nrd4233 24981364 PMC4426976

[B21] Kallakury B. V. Karikehalli S. Haholu A. Sheehan C. E. Azumi N. Ross J. S. (2001). Increased expression of matrix metalloproteinases 2 and 9 and tissue inhibitors of metalloproteinases 1 and 2 correlate with poor prognostic variables in renal cell carcinoma. Clin. Cancer Res. 7, 3113–3119. 11595703

[B22] Kawakami A. Fisher D. E. (2017). The master role of microphthalmia-associated transcription factor in melanocyte and melanoma biology. Lab. Invest. 97, 649–656. 10.1038/labinvest.2017.9 28263292

[B23] Kinoshita S. Inoue Y. Nakama S. Ichiba T. Aniya Y. (2007). Antioxidant and hepatoprotective actions of medicinal herb, Terminalia catappa L. from Okinawa Island and its tannin corilagin. Phytomedicine 14, 755–762. 10.1016/j.phymed.2006.12.012 17293097

[B24] Kloucek P. Polesny Z. Svobodova B. Vlkova E. Kokoska L. (2005). Antibacterial screening of some Peruvian medicinal plants used in Calleria District. J. Ethnopharmacol. 99, 309–312. 10.1016/j.jep.2005.01.062 15894143

[B25] Lamouille S. Xu J. Derynck R. (2014). Molecular mechanisms of epithelial-mesenchymal transition. Nat. Rev. Mol. Cell Biol. 15, 178–196. 10.1038/nrm3758 24556840 PMC4240281

[B26] Larue L. Delmas V. (2006). The WNT/Beta-catenin pathway in melanoma. Front. Biosci. 11, 733–742. 10.2741/1831 16146765

[B27] Levy C. Khaled M. Fisher D. E. (2006). Mitf: Master regulator of melanocyte development and melanoma oncogene. Trends Mol. Med. 12, 406–414. 10.1016/j.molmed.2006.07.008 16899407

[B28] Liu X. Feng R. (2010). Inhibition of epithelial to mesenchymal transition in metastatic breast carcinoma cells by c-Src suppression. Acta Biochim. Biophys. Sin. 42, 496–501. 10.1093/abbs/gmq043 20705589

[B29] Lo J. A. Fisher D. E. (2014). The melanoma revolution: From UV carcinogenesis to a new era in therapeutics. Science 346, 945–949. 25414302 10.1126/science.1253735 PMC4701046

[B30] Maziveyi M. Alahari S. K. (2017). Cell matrix adhesions in cancer: The proteins that form the glue. Oncotarget 8, 48471–48487. 10.18632/oncotarget.17265 28476046 PMC5564663

[B31] Mitra S. K. Schlaepfer D. D. (2006). Integrin-regulated FAK-Src signaling in normal and cancer cells. Curr. Opin. Cell Biol. 18, 516–523. 10.1016/j.ceb.2006.08.011 16919435

[B32] Molenaar M. van de Wetering M. Oosterwegel M. Peterson-Maduro J. Godsave S. Korinek V. (1996). XTcf-3 transcription factor mediates beta-catenin-induced axis formation in Xenopus embryos. Cell 86, 391–399. 10.1016/s0092-8674(00)80112-9 8756721

[B33] Mosmann T. (1983). Rapid colorimetric assay for cellular growth and survival: Application to proliferation and cytotoxicity assays. J. Immunol. Methods 65, 55–63. 10.1016/0022-1759(83)90303-4 6606682

[B34] Mousson A. Legrand M. Steffan T. Vauchelles R. Carl P. Gies J. P. (2021). Inhibiting FAK-paxillin interaction reduces migration and invadopodia-mediated matrix degradation in metastatic melanoma cells. Cancers (Basel) 13, 1871. 10.3390/cancers13081871 33919725 PMC8070677

[B35] Nader G. P. Ezratty E. J. Gundersen G. G. (2016). FAK, talin and PIPKIγ regulate endocytosed integrin activation to polarize focal adhesion assembly. Nat. Cell Biol. 18, 491–503. 10.1038/ncb3333 27043085

[B36] Nagathihalli N. S. Merchant N. B. (2012). Src-mediated regulation of E-cadherin and EMT in pancreatic cancer. Front. Biosci. 17, 2059–2069. 10.2741/4037 22652764

[B37] Naitik P. Prakash T. Kotresha D. Rao N. R. (2012). Effect of Terminalia catappa on lipid profile in transplanted fibrosarcoma in rats. Indian J. Pharmacol. 44, 390–392. 10.4103/0253-7613.96345 22701253 PMC3371466

[B38] Pandya N. B. Tigari P. Dupadahalli K. Kamurthy H. Nadendla R. R. (2013). Antitumor and antioxidant status of Terminalia catappa against Ehrlich ascites carcinoma in Swiss albino mice. Indian J. Pharmacol. 45, 464–469. 10.4103/0253-7613.117754 24130380 PMC3793516

[B39] Parsons J. T. (2003). Focal adhesion kinase: The first ten years. J. Cell Sci. 116, 1409–1416. 10.1242/jcs.00373 12640026

[B40] Ring A. Kim Y. M. Kahn M. (2014). Wnt/catenin signaling in adult stem cell physiology and disease. Stem Cell Rev. Rep. 10, 512–525. 10.1007/s12015-014-9515-2 24825509 PMC4294579

[B41] Sandru A. Voinea S. Panaitescu E. Blidaru A. (2014). Survival rates of patients with metastatic malignant melanoma. J. Med. Life 7, 572–576. 25713625 PMC4316142

[B42] Serrels A. Canel M. Brunton V. G. Frame M. C. (2011). Src/FAK-mediated regulation of E-cadherin as a mechanism for controlling collective cell movement: Insights from *in vivo* imaging. Cell adh. Migr. 5, 360–365. 10.4161/cam.5.4.17290 21836391 PMC3210304

[B43] Shang N. Arteaga M. Zaidi A. Stauffer J. Cotler S. J. Zeleznik-Le N. J. (2015). FAK is required for c-Met/β-catenin-driven hepatocarcinogenesis. Hepatology 61, 214–226. 10.1002/hep.27402 25163657 PMC4280291

[B44] Shang N. Wang H. Bank T. Perera A. Joyce C. Kuffel G. (2019). Focal adhesion kinase and beta-catenin cooperate to induce hepatocellular carcinoma. Hepatology 70, 1631–1645. 10.1002/hep.30707 31069844 PMC6819211

[B45] Shinde S. L. Junne S. B. Wadje S. S. Baig M. M. (2009). The diversity of antibacterial compounds of Terminalia species (Combretaceae). Pak. J. Biol. Sci. 12, 1483–1486. 10.3923/pjbs.2009.1483.1486 20180323

[B46] Simoes M. C. F. Sousa J. J. S. Pais A. (2015). Skin cancer and new treatment perspectives: A review. Cancer Lett. 357, 8–42. 10.1016/j.canlet.2014.11.001 25444899

[B47] Skinner H. D. Giri U. Yang L. Woo S. H. Story M. D. Pickering C. R. (2016). Proteomic profiling identifies PTK2/FAK as a driver of radioresistance in HPV-negative head and neck cancer. Clin. Cancer Res. 22, 4643–4650. 10.1158/1078-0432.CCR-15-2785 27036135 PMC5061056

[B48] Stearns M. Stearns M. E. (1996). Evidence for increased activated metalloproteinase 2 (MMP-2a) expression associated with human prostate cancer progression. Oncol. Res. 8, 69–75. 8859777

[B49] Stetler-Stevenson W. G. (1999). Matrix metalloproteinases in angiogenesis: A moving target for therapeutic intervention. J. Clin. Invest. 103, 1237–1241. 10.1172/JCI6870 10225966 PMC408361

[B50] Sulzmaier F. J. Jean C. Schlaepfer D. D. (2014). FAK in cancer: Mechanistic findings and clinical applications. Nat. Rev. Cancer 14, 598–610. 10.1038/nrc3792 25098269 PMC4365862

[B51] Talvensaari-Mattila A. Paakko P. Hoyhtya M. Blanco-Sequeiros G. Turpeenniemi-Hujanen T. (1998). Matrix metalloproteinase-2 immunoreactive protein: A marker of aggressiveness in breast carcinoma. Cancer 83, 1153–1162. 10.1002/(sici)1097-0142(19980915)83:6<1153::aid-cncr14>3.0.co;2-4 9740080

[B52] Tan G. T. Pezzuto J. M. Kinghorn A. D. Hughes S. H. (1991). Evaluation of natural products as inhibitors of human immunodeficiency virus type 1 (HIV-1) reverse transcriptase. J. Nat. Prod. 54, 143–154. 10.1021/np50073a012 1710653

[B53] Tarapore R. S. Siddiqui I. A. Mukhtar H. (2012). Modulation of Wnt/β-catenin signaling pathway by bioactive food components. Carcinogenesis 33, 483–491. 10.1093/carcin/bgr305 22198211 PMC3384069

[B54] Uzdensky A. B. Demyanenko S. V. Bibov M. Y. (2013). Signal transduction in human cutaneous melanoma and target drugs. Curr. Cancer Drug Targets 13, 843–866. 10.2174/1568009611313080004 23675881

[B55] Volkovova K. Bilanicova D. Bartonova A. Letasiova S. Dusinska M. (2012). Associations between environmental factors and incidence of cutaneous melanoma. Review. Environ. Health 11 (1), S12. 10.1186/1476-069X-11-S1-S12 22759494 PMC3388446

[B56] Westermarck J. Kahari V. M. (1999). Regulation of matrix metalloproteinase expression in tumor invasion. FASEB J. 13, 781–792. 10.1096/fasebj.13.8.781 10224222

[B57] Widlund H. R. Horstmann M. A. Price E. R. Cui J. Lessnick S. L. Wu M. (2002). Beta-catenin-induced melanoma growth requires the downstream target Microphthalmia-associated transcription factor. J. Cell Biol. 158, 1079–1087. 10.1083/jcb.200202049 12235125 PMC2173224

[B58] Worthmuller J. Ruegg C. (2020). The crosstalk between FAK and Wnt signaling pathways in cancer and its therapeutic implication. Int. J. Mol. Sci. 21, E9107. 10.3390/ijms21239107 PMC7730291 33266025

[B59] Yang S. F. Chen M. K. Hsieh Y. S. Yang J. S. Zavras A. I. Hsieh Y. H. (2010). Antimetastatic effects of Terminalia catappa L. on oral cancer via a down-regulation of metastasis-associated proteases. Food Chem. Toxicol. 48, 1052–1058. 10.1016/j.fct.2010.01.019 20102732

[B60] Yang S. F. Yang W. E. Chang H. R. Chu S. C. Hsieh Y. S. (2008). Luteolin induces apoptosis in oral squamous cancer cells. J. Dent. Res. 87, 401–406. 10.1177/154405910808700413 18362328

[B61] Yeh C. B. Hsieh M. J. Hsieh Y. S. Chien M. H. Lin P. Y. Chiou H. L. (2012). Terminalia catappa exerts antimetastatic effects on hepatocellular carcinoma through transcriptional inhibition of matrix metalloproteinase-9 by modulating NF-κB and AP-1 activity. Evid. Based. Complement. Altern. Med. 2012, 595292. 10.1155/2012/595292 PMC3522499 23258989

[B62] Yeh C. B. Yu Y. L. Lin C. W. Chiou H. L. Hsieh M. J. Yang S. F. (2014). Terminalia catappa attenuates urokinase-type plasminogen activator expression through Erk pathways in Hepatocellular carcinoma. BMC Complement. Altern. Med. 14, 141. 10.1186/1472-6882-14-141 24886639 PMC4012530

[B63] Yilmaz M. Christofori G. (2009). EMT, the cytoskeleton, and cancer cell invasion. Cancer Metastasis Rev. 28, 15–33. 10.1007/s10555-008-9169-0 19169796

[B64] Yoshizaki T. Maruyama Y. Sato H. Furukawa M. (2001). Expression of tissue inhibitor of matrix metalloproteinase-2 correlates with activation of matrix metalloproteinase-2 and predicts poor prognosis in tongue squamous cell carcinoma. Int. J. Cancer 95, 44–50. 10.1002/1097-0215(20010120)95:1<44::aid-ijc1008>3.0.co;2-m 11241310

