# Corrigendum: *Terminalia catappa* leaf extracts inhibited metastasis of A2058 and A375 melanoma cells *via* downregulating p-Src and β-catenin pathway *in vitro*

**DOI:** 10.3389/fphar.2022.1074693

**Published:** 2022-11-18

**Authors:** Chin-Kuo Chang, Shu-Chen Chu, Jing-Yang Huang, Pei-Ni Chen, Yih-Shou Hsieh

**Affiliations:** ^1^ Institute of Medicine Chung Shan Medical University, Taichung, Taiwan; ^2^ Institute and Department of Food Science Central Taiwan University of Science and Technology, Taichung, Taiwan; ^3^ Department of Medical Research Chung Shan Medical University Hospital, Taichung, Taiwan; ^4^ Department of Biochemistry School of Medicine Chung Shan Medical University, Taichung, Taiwan; ^5^ Clinical Laboratory Chung Shan Medical University Hospital, Taichung, Taiwan

**Keywords:** *Terminalia catappa*, melanoma, antimetastasis, matrix metalloproteinases-2, Wnt/ βcatenin pathways

In the published article, there were two errors in Figures 4, 5. First, the unit of four concentrations in Figures 4A,B were mismarked as “mg/mL.” The correct unit should be “μg/mL” in the quantization map of Figures 4A,B. Second, the wrong images were used in Figures 5A,B (at the concentration of 25 μg/mL). The corrected Figures 4, 5 and their captions appear below.

**FIGURE 4:**
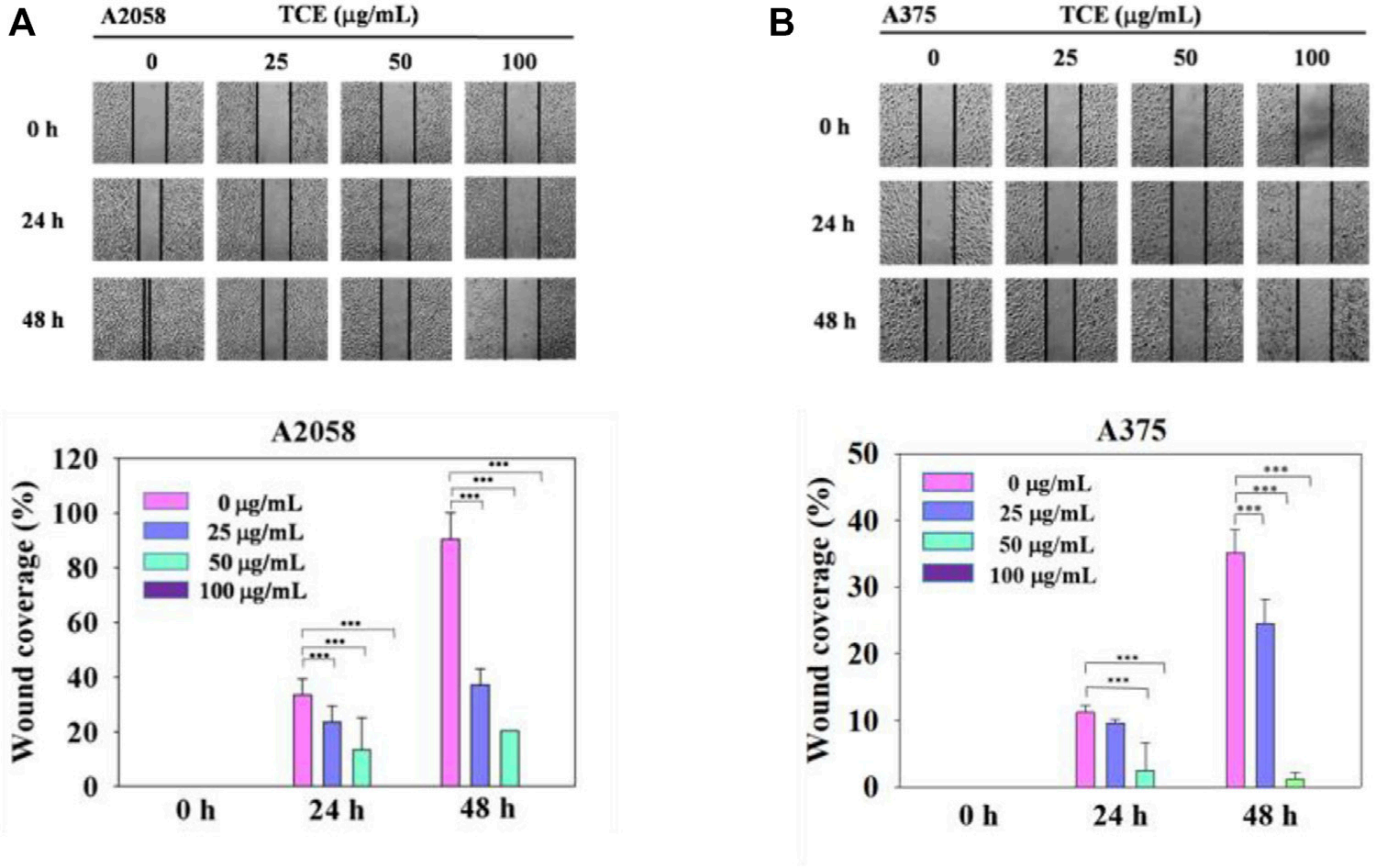
The effects of TCE on the cell migration of A2058 and A375 cells. **(A,B)** Photographs showed wound closure of A2058 and A375 cells treated with TCE (0, 25, 50, 100 μg/mL) and 0, 24, 48 h. Cell wound healing assay was performed as described in the Materials and Methods. % Wound coverage was determined by the rate of cells moving forward the scratched area upon time. The values of percentage wound closure ± SD of at least three independent experiments. Comparisons were performed using two-way ANOVA with post hoc Turkey’s test (***p < 0.05).

**FIGURE 5:**
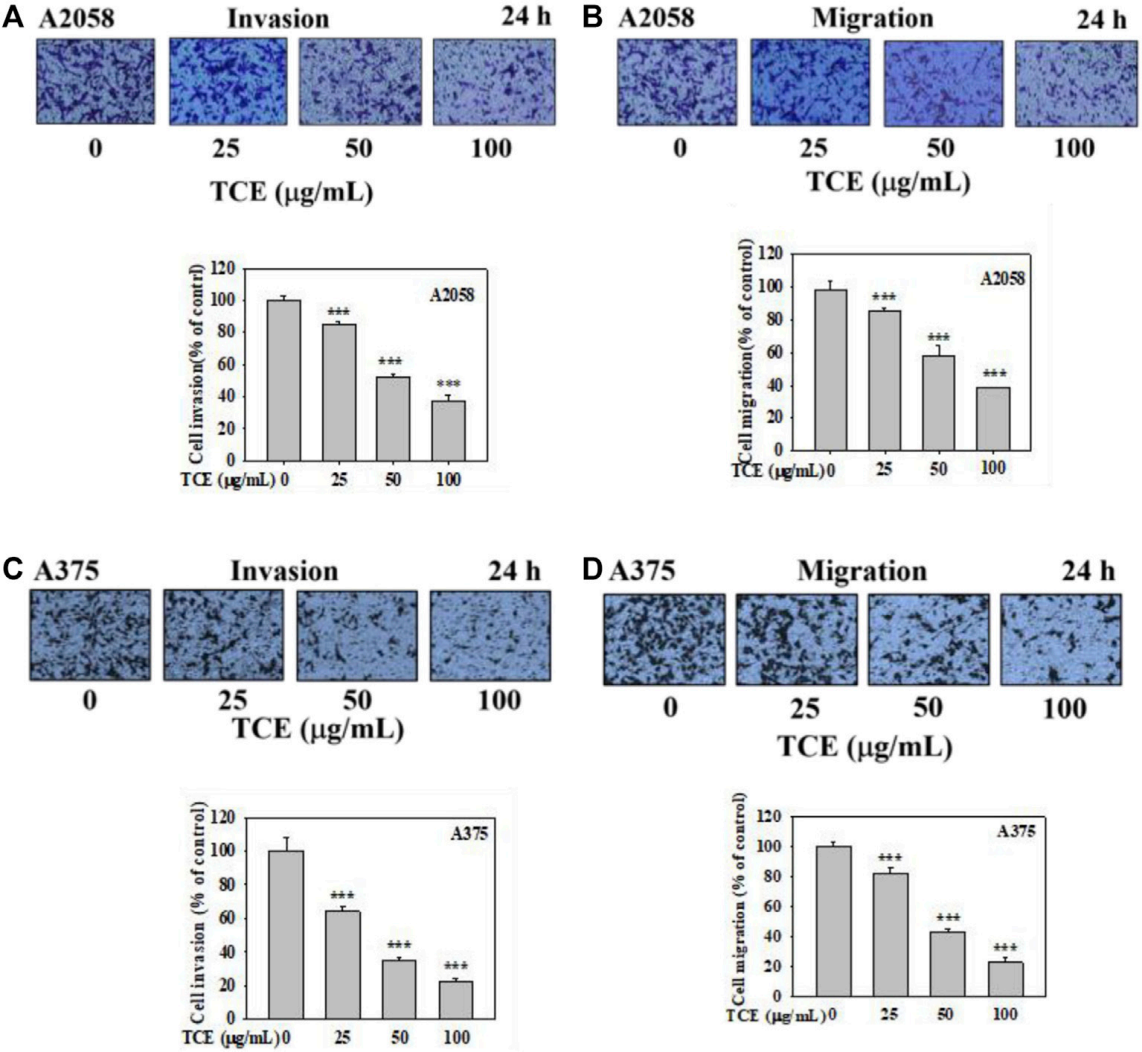
Effects of TCE on cell invasion and migration of A2058 and A375 cells. After being treated with TCE at a concentration of (0, 25, 50, 100 μg/mL) for 24 h, cell invasion and migration assay were then performed as described in the Materials and Methods. Representative images of A2058 and A375 cells on the lower side of membrane at different time points in the **(A,C)** invasion and **(B,D)** migration assay. A representative number of invading cells through the Matrigel and membrane were counted under the microscope for 10 random fields at a 3,200 magnification. The results were statistically evaluated using two-way ANOVA with post hoc Turkey’s test (***p < 0.05). The results from 3 repeated and separate experiments were similar.

The authors apologize for these errors and states that this does not change the scientific conclusions of the article in any way. The original article has been updated.

