# Supplementary figures and images for: Terminalia catappa leaf extracts inhibited metastasis of A2058 and A375 melanoma cells via downregulating p-Src and β-catenin pathway in vitro
Source: Front Pharmacol. 2022 Sep 27;13:963589. doi: 10.3389/fphar.2022.963589 (PMC9551286; doi:10.3389/fphar.2022.963589)

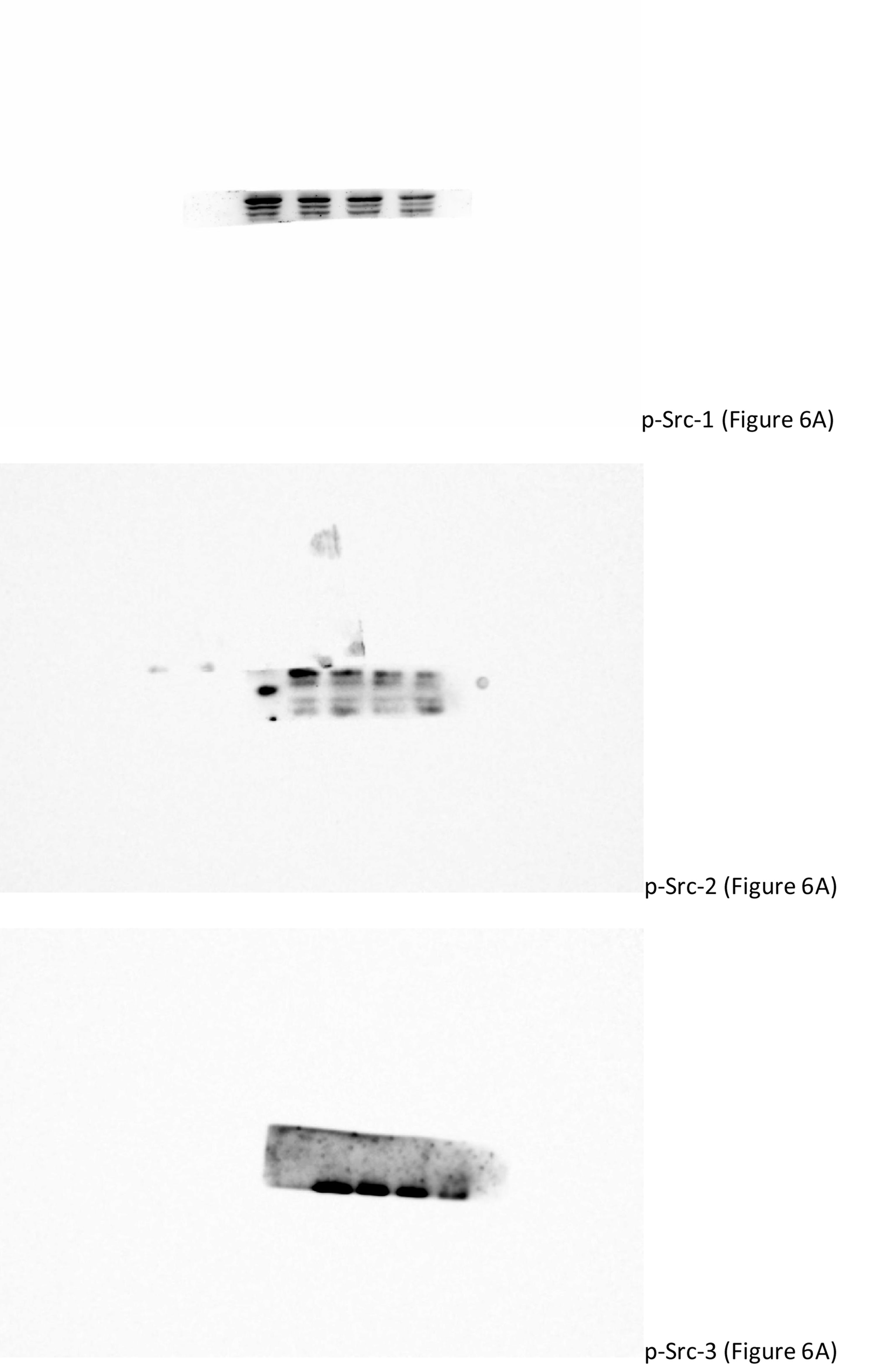

Supplement: Supplementary file 1 [file Image3.JPEG]

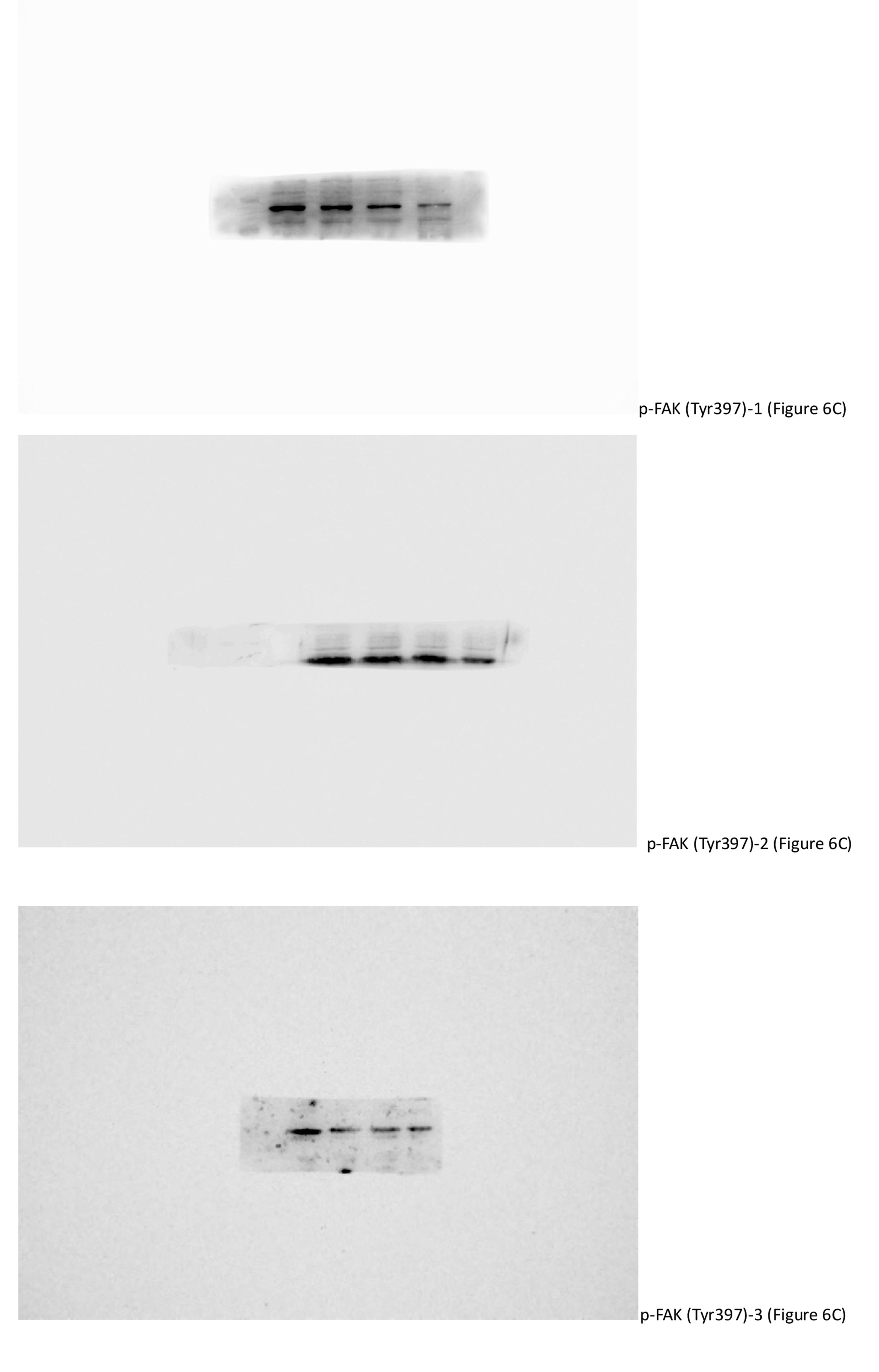

Supplement: Supplementary file 2 [file Image9.JPEG]

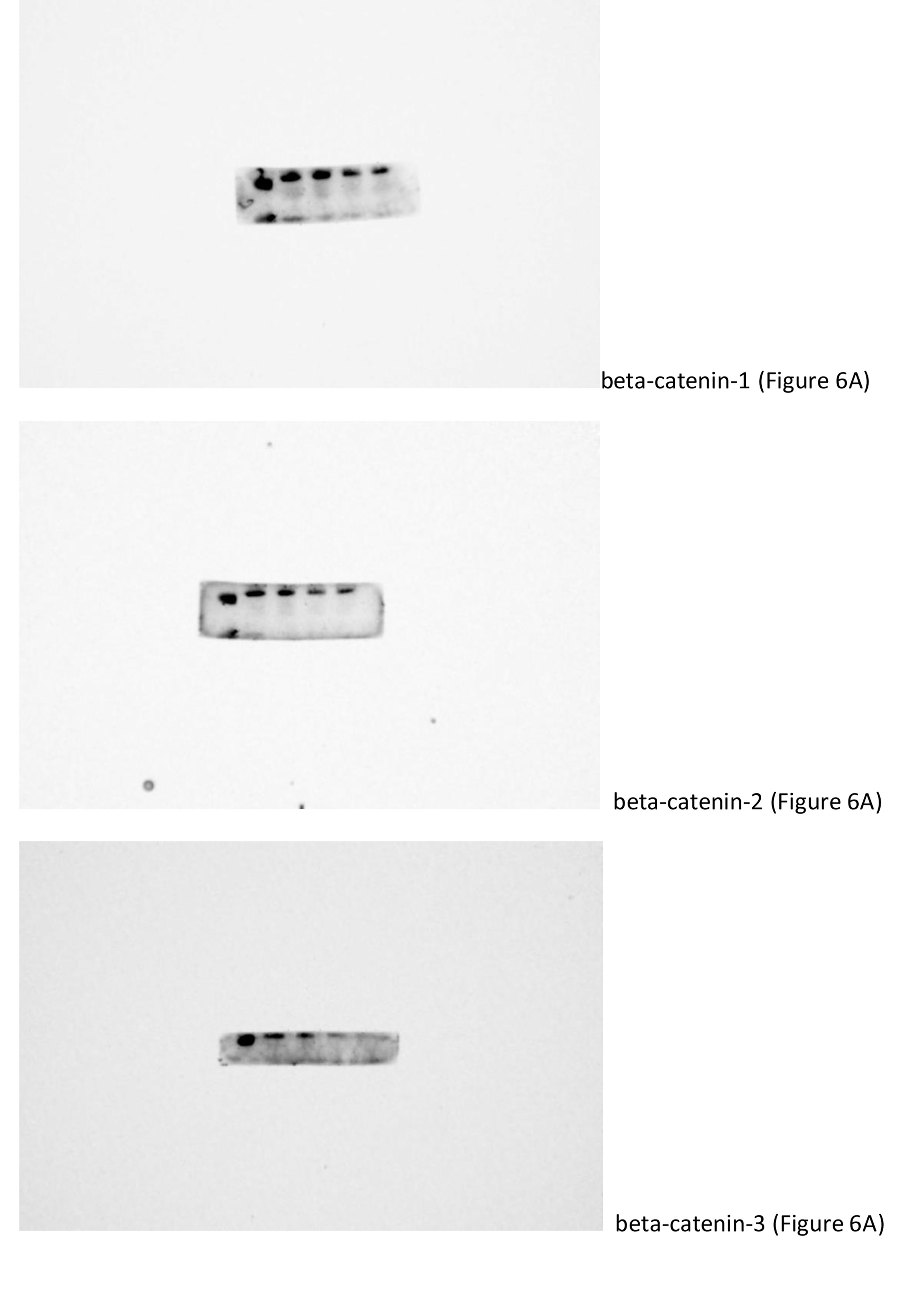

Supplement: Supplementary file 3 [file Image1.JPEG]

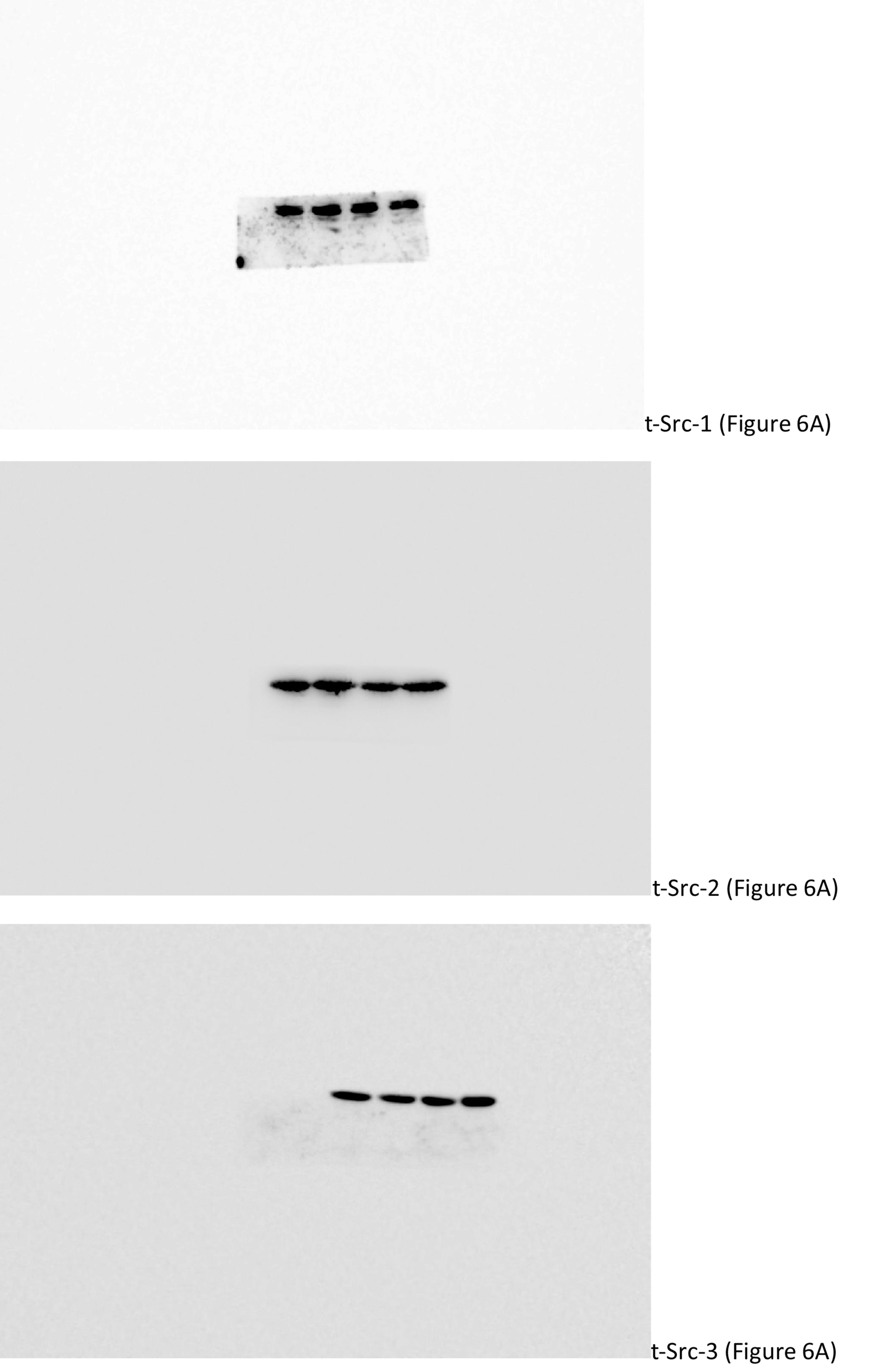

Supplement: Supplementary file 4 [file Image4.JPEG]

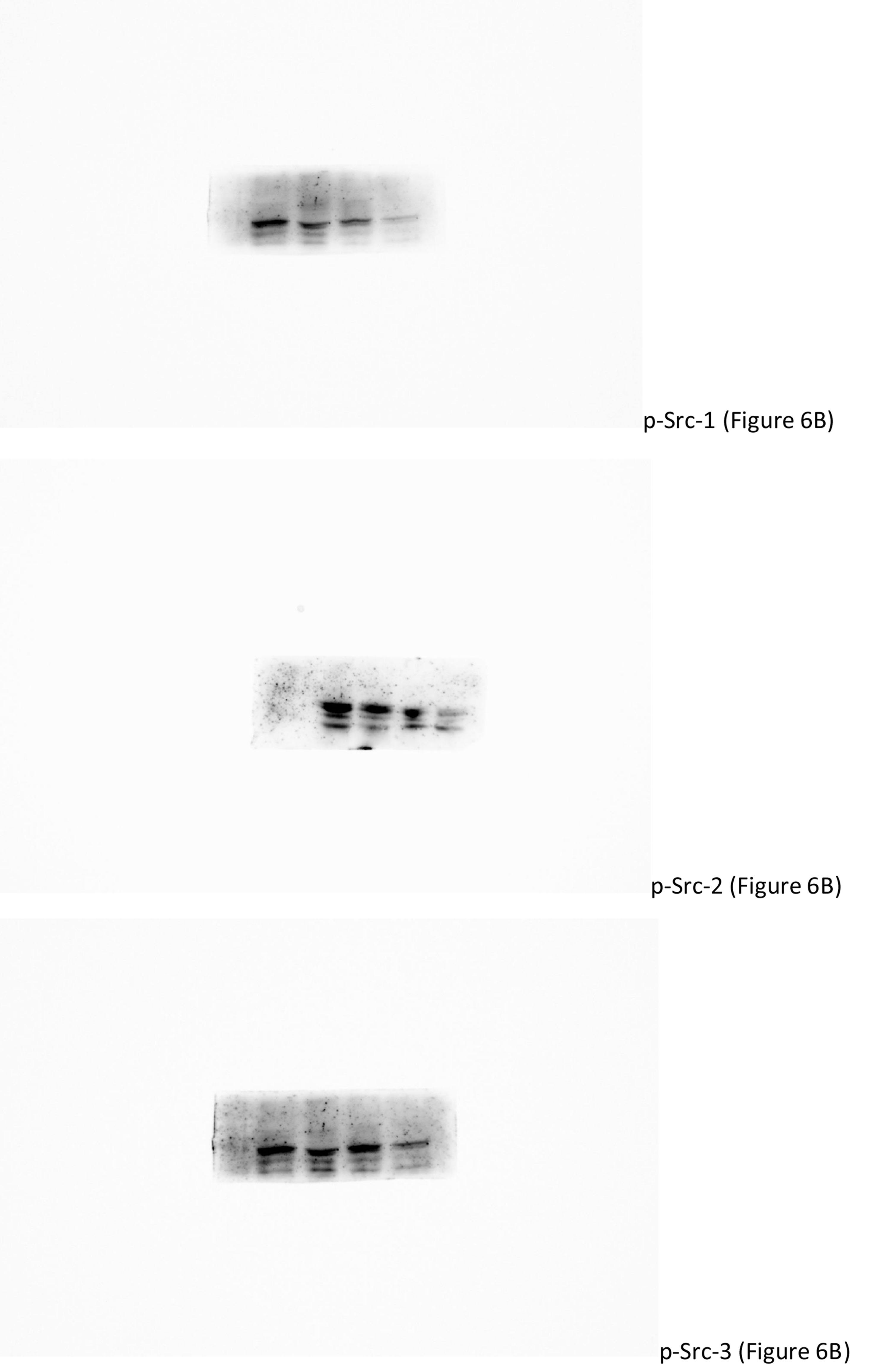

Supplement: Supplementary file 5 [file Image7.JPEG]

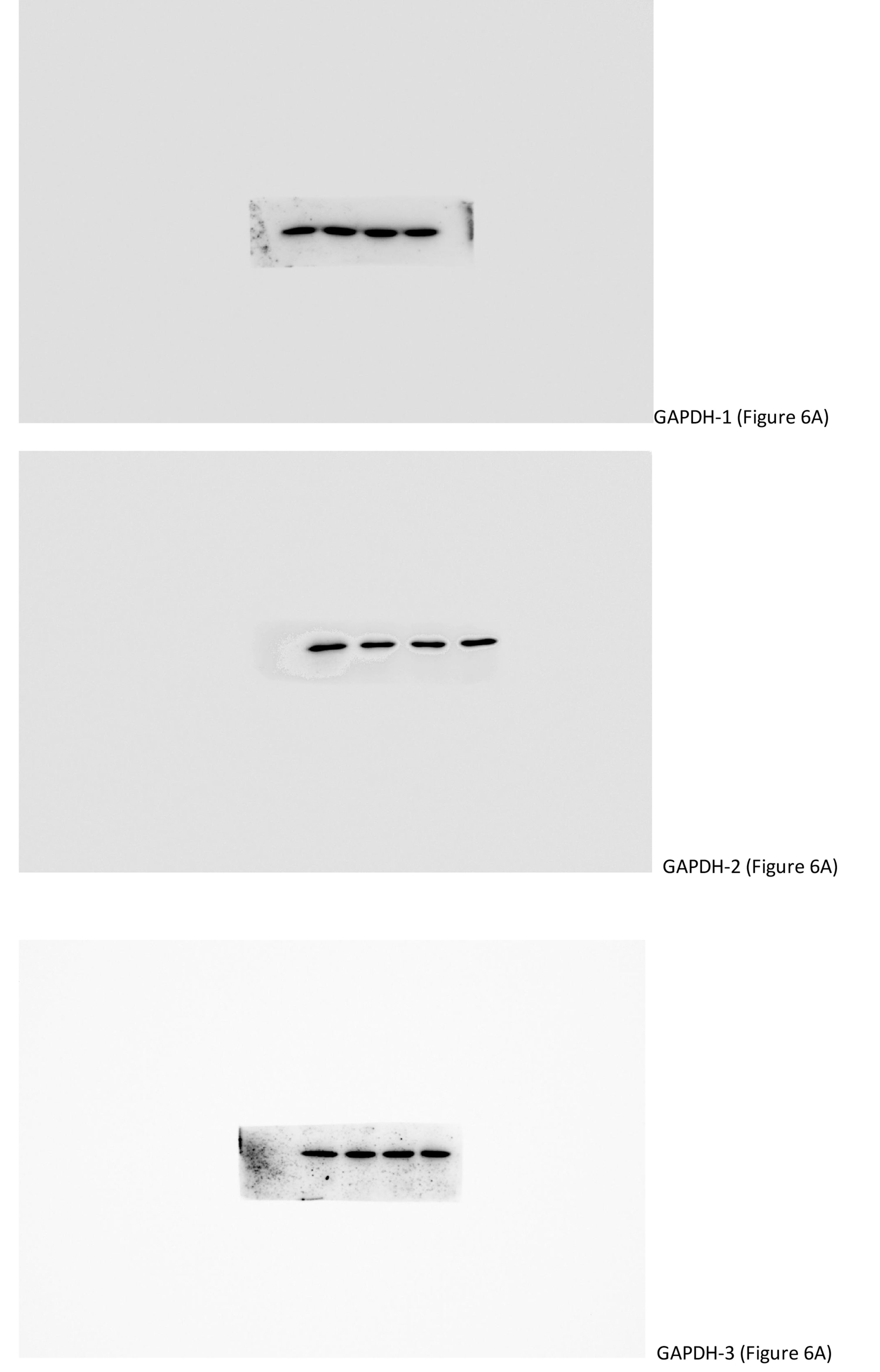

Supplement: Supplementary file 6 [file Image2.JPEG]

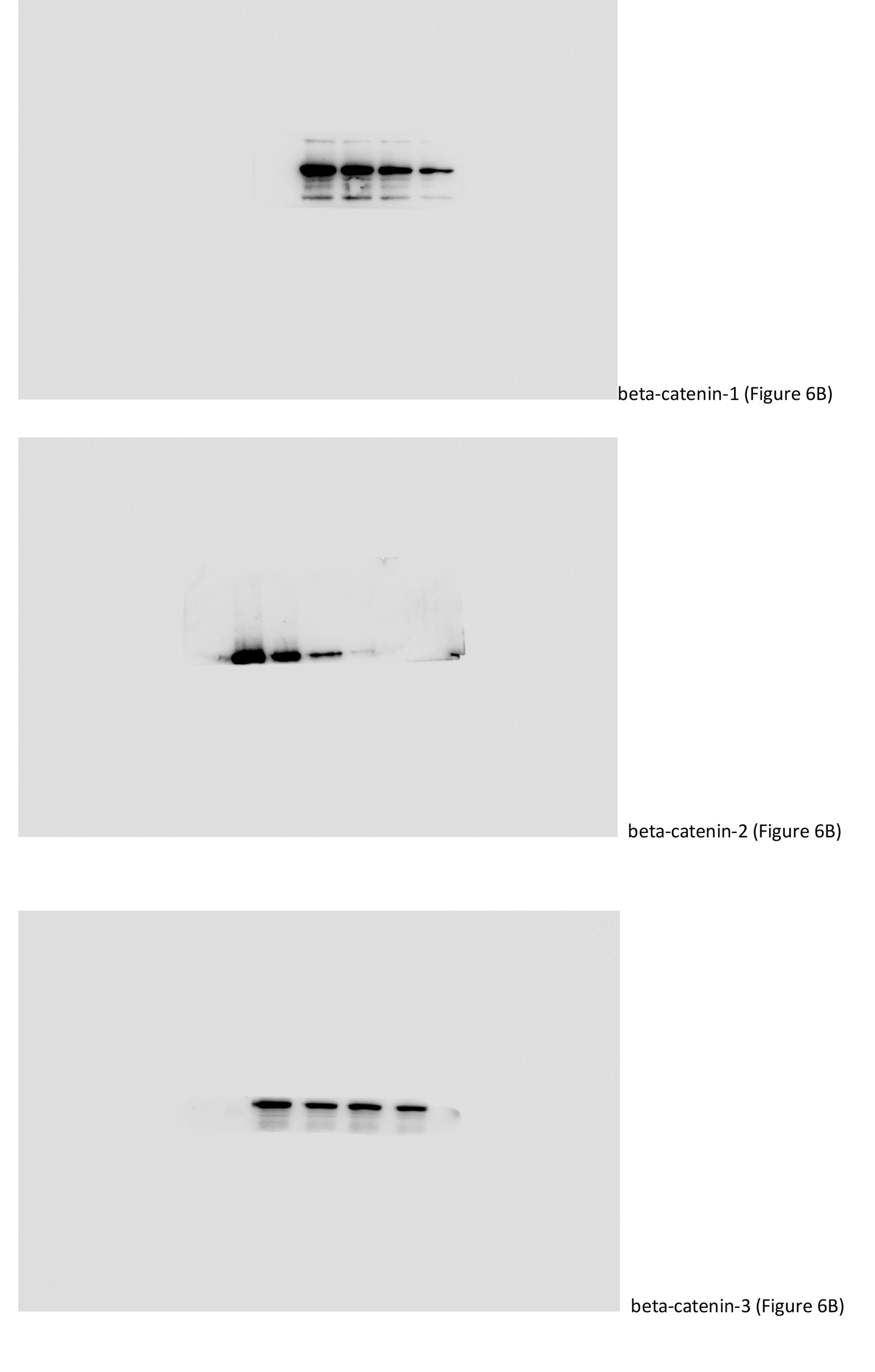

Supplement: Supplementary file 7 [file Image5.JPEG]

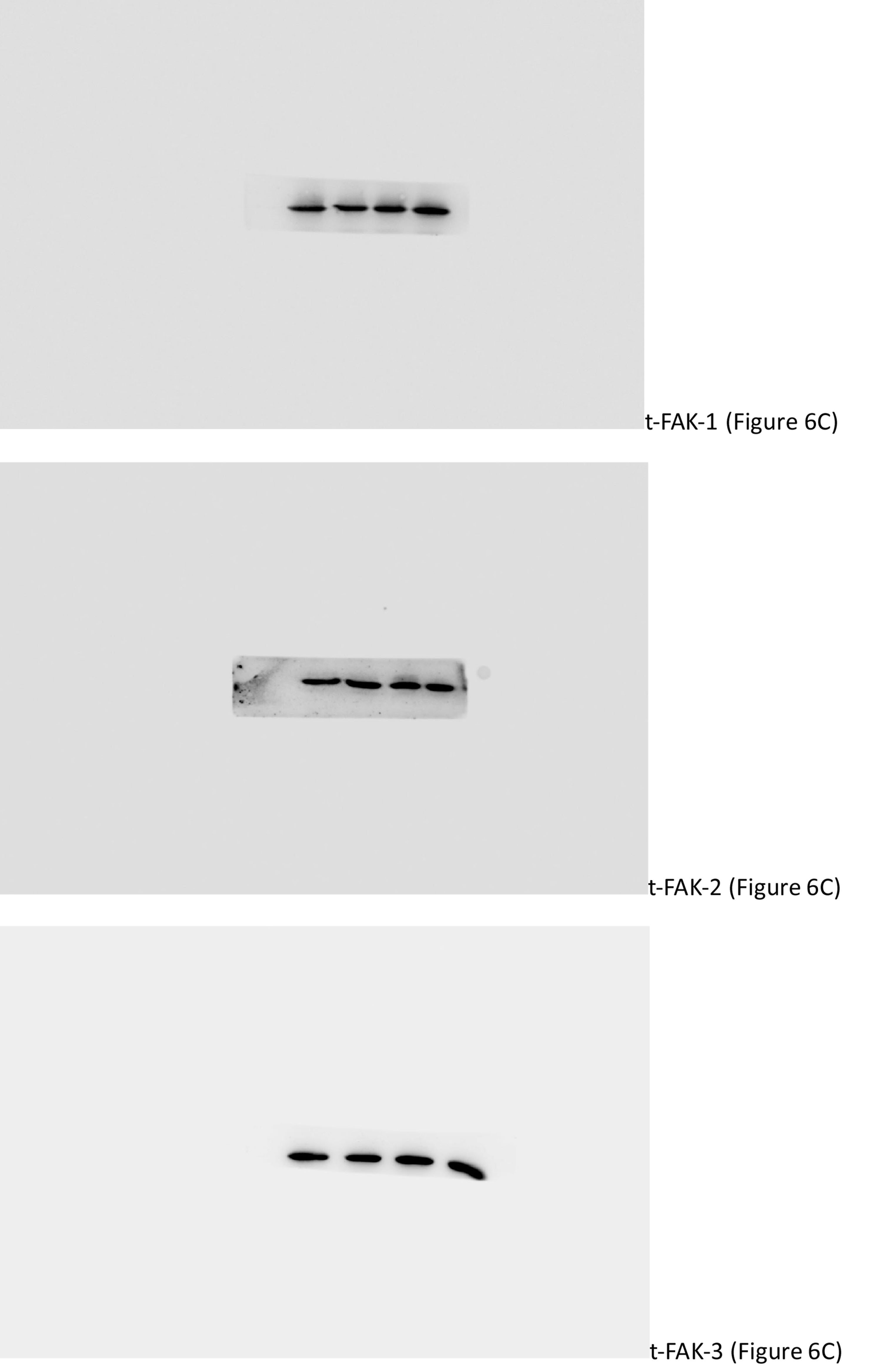

Supplement: Supplementary file 8 [file Image10.JPEG]

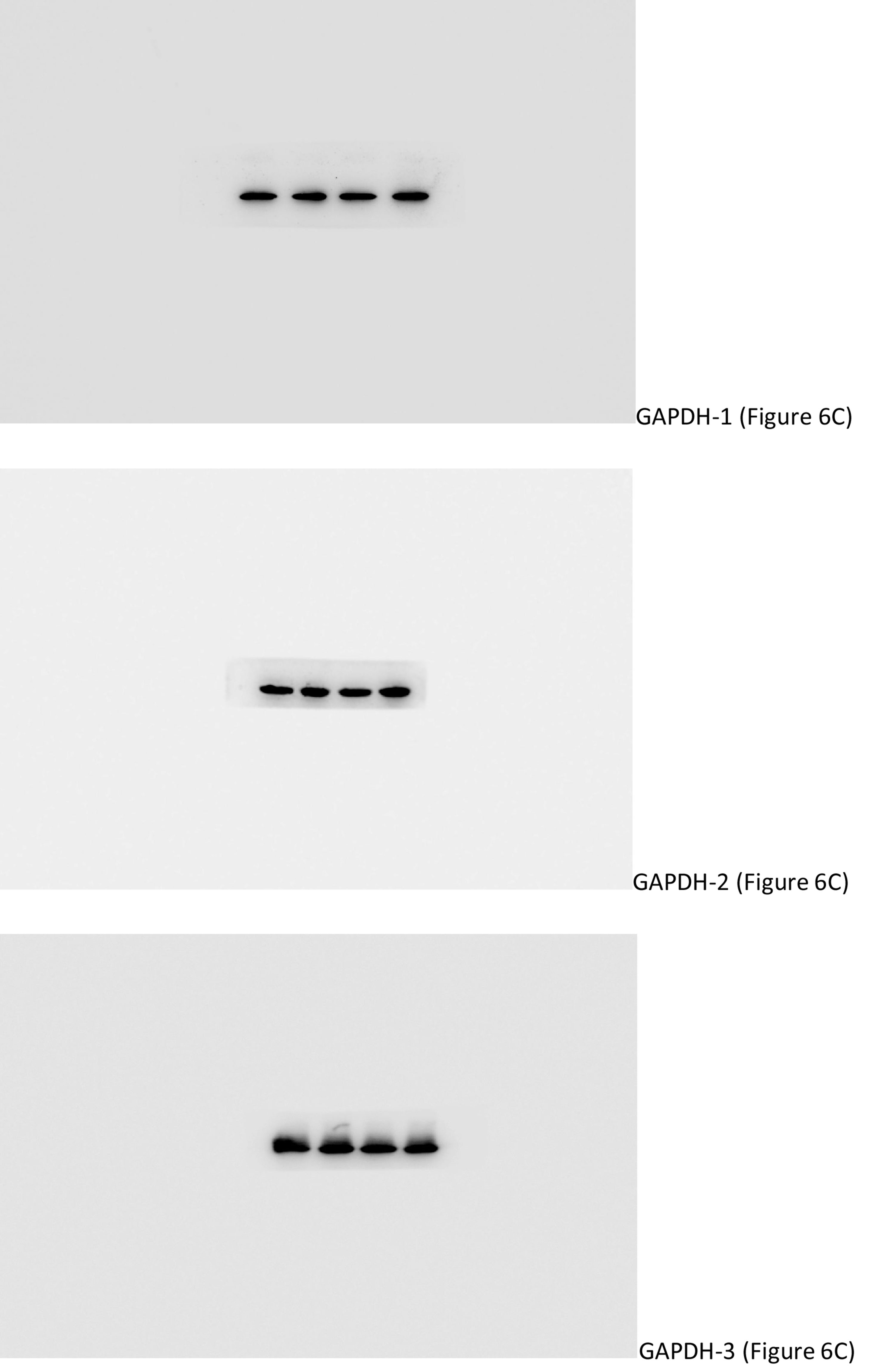

Supplement: Supplementary file 9 [file Image12.JPEG]

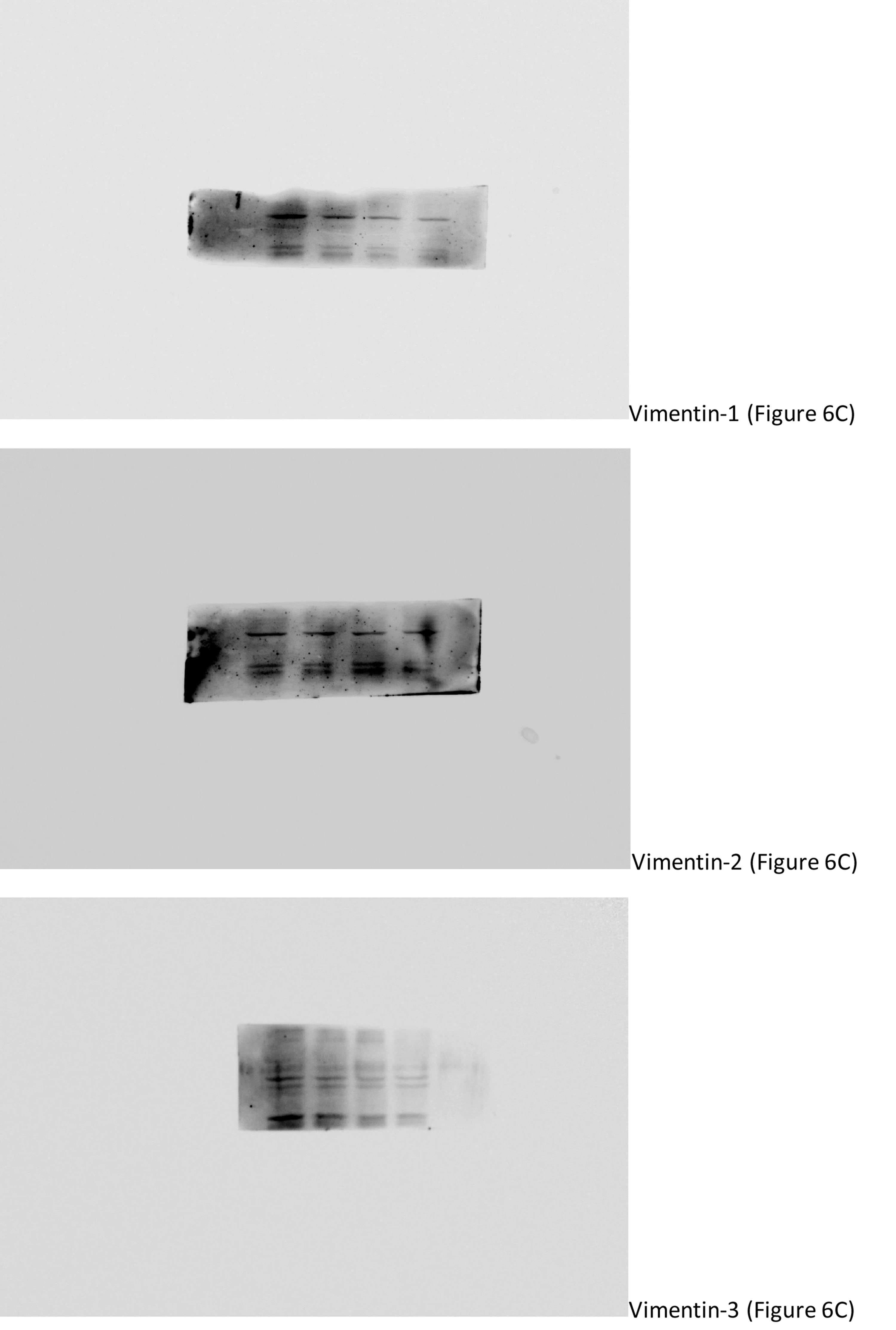

Supplement: Supplementary file 10 [file Image11.JPEG]

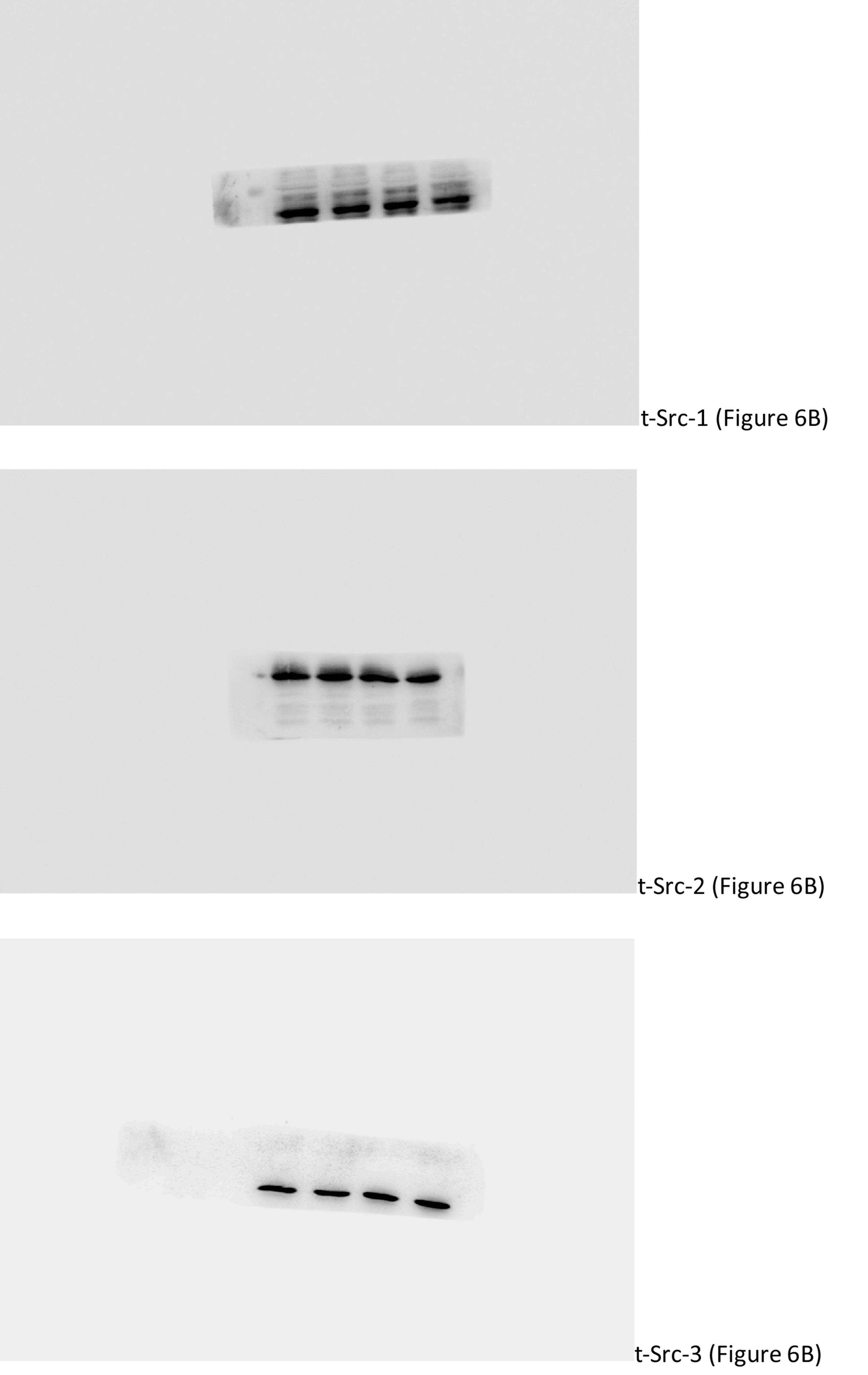

Supplement: Supplementary file 11 [file Image8.JPEG]

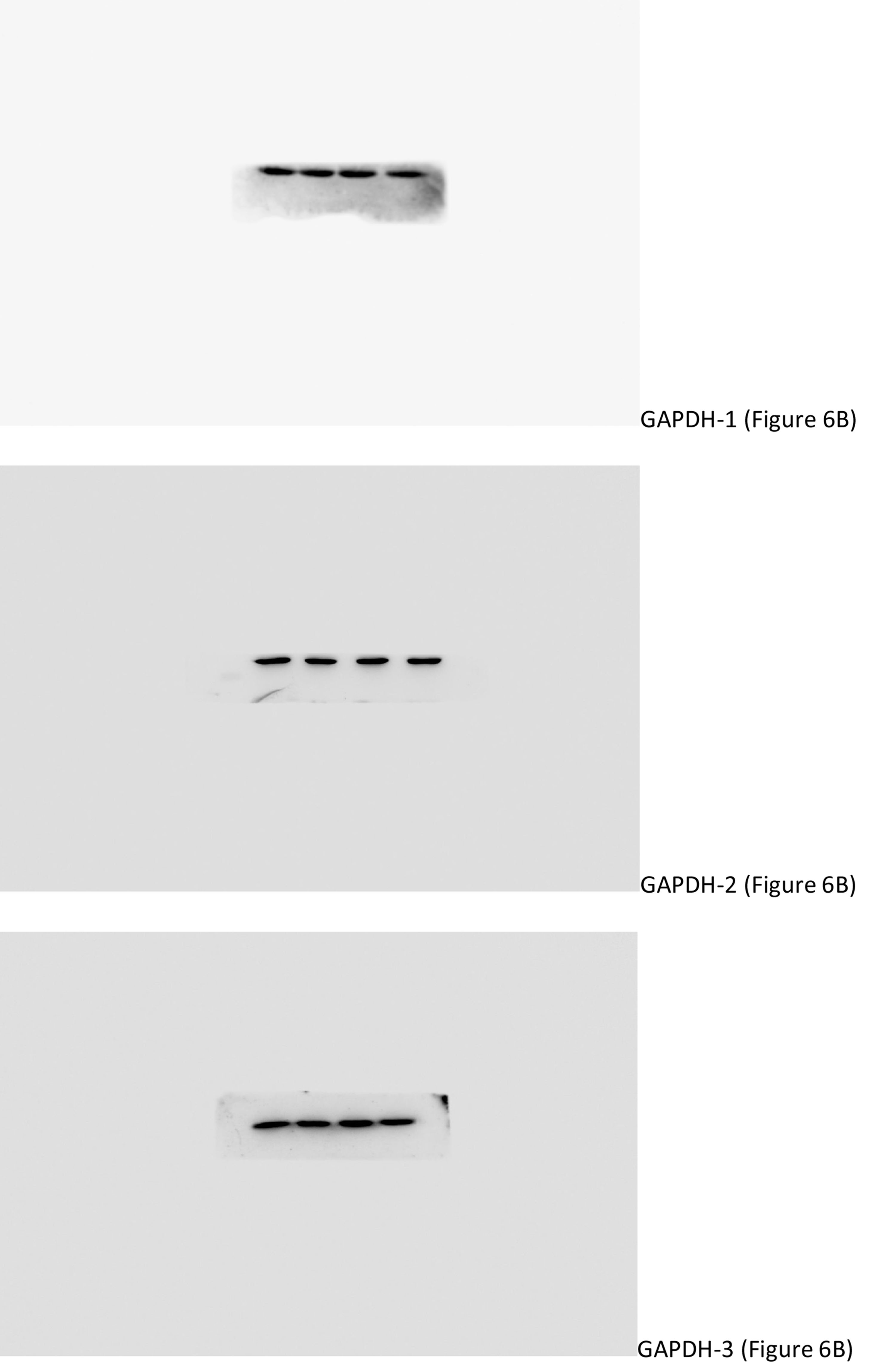

Supplement: Supplementary file 12 [file Image6.JPEG]
